# Danhong Injection Reversed Cardiac Abnormality in Brain–Heart Syndrome via Local and Remote β-Adrenergic Receptor Signaling

**DOI:** 10.3389/fphar.2018.00692

**Published:** 2018-07-03

**Authors:** John O. Orgah, Jiahui Yu, Tiechan Zhao, Lingyan Wang, Mingzhu Yang, Yan Zhang, Guanwei Fan, Yan Zhu

**Affiliations:** ^1^Tianjin State Key Laboratory of Modern Chinese Medicine, Tianjin University of Traditional Chinese Medicine, Tianjin, China; ^2^Research and Development Center of TCM, Tianjin International Joint Academy of Biotechnology and Medicine, Tianjin, China; ^3^First Teaching Hospital of Tianjin University of Traditional Chinese Medicine, Tianjin, China

**Keywords:** brain–heart syndrome, cerebral ischemic stroke, β-adrenergic receptor, cardiac dysfunction, Danhong injection

## Abstract

Ischemic brain injury impacts cardiac dysfunction depending on the part of the brain affected, with a manifestation of irregular blood pressure, arrhythmia, and heart failure. Generally called brain–heart syndrome in traditional Chinese medicine, few mechanistic understanding and treatment options are available at present. We hypothesize that considering the established efficacy for both ischemic stroke and myocardial infarction (MI), Danhong injection (DHI), a multicomponent Chinese patent medicine, may have a dual pharmacological potential for treating the brain–heart syndrome caused by cerebral ischemic stroke through its multi-targeted mechanisms. We investigated the role of DHI in the setting of brain–heart syndrome and determined the mechanism by which it regulates this process. We induced Ischemia/Reperfusion in Wistar rats and administered intravenous dose of DHI twice daily for 14 days. We assessed the neurological state, infarct volume, CT scan, arterial blood pressure, heart rhythm, and the hemodynamics. We harvested the brain and heart tissues for immunohistochemistry and western blot analyses. Our data show that DHI exerts potent anti-stroke effects (infarct volume reduction: ^∗∗^*p* < 0.01 and ^∗∗∗^*p* < 0.001 vs. vehicle. Neurological deficit correction: ^∗^*p* < 0.05 and ^∗∗∗^*p* < 0.001 vs. vehicle), and effectively reversed the abnormal arterial pressure (^∗^*p* < 0.05 vs. vehicle) and heart rhythm (^∗∗^*p* < 0.01 vs. vehicle). The phenotype of this brain–heart syndrome is strikingly similar to those of MI model. Quantitative assessment of hemodynamic in cardiac functionality revealed a positive uniformity in the PV-loop after administration with DHI and valsartan in the latter. Immunohistochemistry and western blot results showed the inhibitory effect of DHI on the β-adrenergic pathway as well as protein kinase C epsilon (PKCε) (^∗∗^*p* < 0.01 vs. model). Our data showed the underlying mechanisms of the brain–heart interaction and offer the first evidence that DHI targets the adrenergic pathway to modulate cardiac function in the setting of brain–heart syndrome. This study has made a novel discovery for proper application of the multi-target DHI and could serve as a therapeutic option in the setting of brain–heart syndrome.

## Introduction

Although stroke and heart disease are the leading cause of disability and death in adults worldwide, there is no effective treatment for acute ischemic stroke apart from thrombolytic agents. Neurological association between acute Cerebral ischemic/reperfusion injury (CI/RI), cardiac autonomic imbalance, and MI has been extensively studied in recent years (Palma and Benarroch, 2014), and authors showed that brain and heart are intrinsically connected through the neuro-axis and neuroendocrine system (Levy and Martin, 1984; Ishikawa et al., 2013). For instance, stimulation of left insular cortex in epileptic patients elicited bradycardia and depressor responses (Oppenheimer et al., 1992). Also, hemispheric stroke is implicated in the dysregulation of cardiac autonomic activity (Hasan, 2013). The recent clinical study showed an association between cryptogenic stroke and arterial fibrillation, which occurs in 30–40% of first-time stroke patients (Wang et al., 2013). Cerebral ischemic stroke and myocardial infarction (MI) have a common pathological pathway and sometimes occurs concomitantly in patients (Kallmünzer et al., 2012), with cardiac injury and arrhythmias being common in patients having cerebrovascular disease, and contribute to mortality in a number of pathological conditions (Kallmünzer et al., 2012). Evidence suggests that brain, heart, and kidney interacts sequel to CI/RI, and cause long-lasting disruptive effect on the cardiovascular autonomic regulatory system resulting in cardiac dysfunction due to impaired function of the sympathetic and parasympathetic tone mediated by norepinephrine and acting via β-adrenergic receptors (β-ARs) (Ishikawa et al., 2013; Palma and Benarroch, 2014; Silvani et al., 2016). During CI/RI, there is an abnormal release of neurotransmitters and inflammatory cytokines among others when neurochemicals bind to receptors (Sanchez-Mendoza et al., 2012), a neurohormonal mechanism in which the adrenergic nervous system plays an active role, causing hyperactivity/toxicity due to its accumulation of binding to ARs. β-ARs have long been quantified in both human and animal’s brain (Sscarpace and Abrass, 1988) and heart (Port and Bristow, 2001). β-ARs are activated by adrenaline and noradrenaline. β_1_-AR dominates in the non-failing human heart, with a greater binding affinity of the neurotransmitter (Port and Bristow, 2001). Heart failure (HF), as well as CI/RI-induced HF, is linked with the β-AR levels in the human ventricular myocardium.

Several clinical and experimental data have implicated the failure of the blood–brain barrier (BBB) during CI/RI (Kumar et al., 2009) and indirectly links β-ARs accumulation through systemic inflammation (Chen et al., 2017). Activation of toll-like receptors by damage-associated molecular patterns including pro-inflammatory cytokines consequent to brain damage induces HF through series of mechanisms which in turn promotes the accumulation of β-ARs toxicity resulting in myocardial death (Kizaki et al., 2008).

The therapeutic strategies aimed at restoring blood flow to ischemic brain called reperfusion is frequently used in clinical practice (Molina and Saver, 2005). Moreover, potential neuroprotective therapies aimed at preventing reperfusion injury and protect BBB dysfunction, salvage penumbra neurons to achieve early neurologic recovery and to prevent cardiac dysfunction (Pan et al., 2007).

Since the brain–heart connection is multi-faceted, it requires a multi-component medicine such as Danhong injection (DHI) (made from the extracts of Radix *Salvia miltiorrhizae* and *Flos Carthami tinctorii*) which have the advantage of multi-targets in various disease network and pathways (He et al., 2012). We have previously identified 11 polyphenolic acids in DHI using ultra-performance liquid chromatography coupled with UV detection (Liu et al., 2013). With a newly developed proton nuclear magnetic resonance profiling method, we also simultaneously quantified 23 primary metabolites together with 7 polyphenolic acids in DHI (Jiang et al., 2014). Recently, other investigators have further identified a total of 63 compounds, including 33 phenolic acids, 2 C-glycosyl quinochalcones, 6 flavonoid *O*-glycosides, 4 iridoid glycosides, 6 organic acids, 5 amino acids, and 3 nucleosides in DHI (Eid et al., 2016; Zhang et al., 2016). We previously showed that DHI reduces vascular remodeling and up-regulates the Kallikrein-kinin system in spontaneously hypertensive rats (Yang et al., 2017). We also found salvianolic acids as core active ingredients of DHI for treating arterial thrombosis and its derived dry gangrene (Zhao et al., 2017). Interestingly, we found DHI protected rat cardiac myocyte and neuronal cells damage induced by overdose arginine vasopressin (Yang et al., 2016). Finally, DHI is notable for its effects on microcirculatory dysfunction (Han et al., 2017).

Although several investigations on the role of DHI in CI/RI and MI had been performed separately in the past, the impact on brain ischemic injury induced cardiac dysfunction, i.e., in a setting of the brain–heart syndrome, have not been investigated. In this present research, we used a rat model of left CI/RI and MI to study the therapeutic effects on protection, recovering of brain–heart function, and reveal the underlying molecular mechanisms via β-ARs signaling.

## Materials and Methods

In this section including the schematic diagram of the experimental plan is available in the Supplementary Material Online.

### Chemicals and Reagents

Danhong injection (Batch No. 12081024077, 10 mL/ampulla) comprising 750 g Salvia miltiorrhiza, 250 g Safflower, and 7 g Sodium chloride was supplied by Heze Buchang Pharmaceutical, Co., Ltd., China. Minocycline (CAS No. 13614-98-7), urethane (Lot # BCBJ7149V), and 2, 3, 5-tryphenyltetrazolium chloride (TTC, Lot # 129K1867V) were all purchased from Sigma-Aldrich, Inc. (St. Louis, MO, United States). Isoflurane (Lot No. B506) was purchased from Ruiwode Company Shenzhen, Guangzhou China. Valsartan (Batch No. X1651) was purchased from Beijing Novartis Pharm Co., Ltd., Beijing China. Anti-β 1, 2, and 3 ARs were purchased from Abcam. Fetal bovine serum (FBS) was purchased from Gibco, China. Omnipaque (Batch No. 11384882) was purchased from GE Healthcare Ireland Cork, Ireland. Positive control drug minocycline and valsartan was freshly prepared in saline before each experiment. 10 mg/mL concentration of minocycline was made and administered at a dose of 20 mg/kg. Also, 1 mg/mL concentration of valsartan was made and administered at a dose of 10 mg/kg intragastrically.

### Experimental Animals

A total of 150 healthy adult male Wistar rats weighing 250–300 g were obtained from Na RuiZeng experimental animal center (Tianjin, China) for CI/RI and IM model. This study was carried out in accordance with the recommendations in the Guideline for the Care and Use of Laboratory Animals issued by the Ministry of Science and Technology of China (Permit Nos. TCM-LAEC2014004 and TJU20160024). Rats were housed in a laboratory rat’s cage (22° ± 2°C and humidity of 40 ± 5%, under a 12 h light/dark cycle), and received standard chow and water *ad libitum*. Before the experiment, animals were fasted for 12 h, but given free access to water. The experimental procedures were according to the European Union adopted Directive 2010/63/EU, and all animal experiments were performed in accord with the international regulations, following the guidelines of Tianjin University of TCM Animal Research Committee (TCM-LAEC2014004) and approved by the animal care and use committee of Tianjin International Joint Academy of Biotechnology (No. TJU20160021).

### Animal Grouping and Drug Treatment

Rats were randomly divided into six different groups (Sham, Model, Positive control, and DHI-low, -middle, and -high). Three doses of DHI comprising 0.75, 1.5, and 3.0 mL/Kg were selected. 1 h before Middle Cerebral Artery Occlusion (MCAO), rats were pre-treated with DHI intravenously (i.v.) and follow up subsequent twice daily for 14 days. 3.0 mL/Kg normal saline and 20 mg/kg minocycline were similarly administered for negative and positive controls, respectively. For MI, the rat model was randomized into three groups: model, valsartan (10 mg/kg), and DHI (0.75 ml/kg). Sham and Model groups were orally administered with normal saline for 28 days.

### MCAO Surgical Procedure

Surgery was performed using transient MCAO technique as described elsewhere (Uluç et al., 2011). Briefly, we anesthetized Wistar rats with 5% isoflurane in 69% N_2_O/30% O_2_. We maintained the body temperature at 37°C ± 0.5, followed by a ventral midline incision in the neck and the dissection of the superficial. We also made a blunt incision to expose the external carotid artery (ECA), internal carotid artery (ICA), and common carotid artery (CCA). A 2.2–3.0 cm length of silicon-coated nylon filament was introduced intraluminally at the CCA into the ICA and forwarded until the tip occluded the origin of the MCA. Cerebral blood flow was monitored using PeriFlux System 5000 during artery occlusion, and ≥80% reduction in regional cerebral blood flow was achieved. For reperfusion to occur, the mono-filament suture was carefully withdrawn after 60 min MCAO, followed by permanent tying of the ECA stump and wound repair. Animals with complications such as excessive bleeding, filament displacement into the pterygopalatine artery, died during or less than 24 h after surgery, were excluded from the experiment.

### Neurological Assessment and Mortality Rate

Infarct size and neurological state of rats were evaluated by three independent observers who were blinded to the surgery. Neurological scores were graded based on specific tasks, using Bederson 4 scale (Bederson et al., 1986). The mortality rate was calculated for the acute period and late time point of CI/RI.

### Quantification of Infarct Volume at Early Time-Point of Reperfusion

Forty-eight hours after CI/RI, three (3) rats were randomly selected from each group and deeply anesthetized with a high dose of isoflurane just before transcardial perfusion with 0.9% normal saline. 2 mm thick coronal sections of the brains and transverse sections of the hearts were obtained and stained in 2, 3, 5-triphenyltetrazolium chloride (TTC). Stained brain and heart sections were captured with a digital camera of high resolution (Leica 14 megapixels Panasonic DMC-FX180). The brain and heart infarct was measured using ImageJ software (Abràmoff et al., 2004) by first of all summing up the infarct areas of the five sections and then presented as the percentage of cerebral ischemic volume in the ipsilateral hemisphere in respect to the total volume and adjusted for edema. Areas of TTC stain defined the healthy region and the unstained hemisphere (ischemic damage defined as core) (Bederson et al., 1986).

### Infarct Volume Quantification at Late Time Point of Reperfusion

At 14th day of the experiment (the end time-point of the study and the last DHI administration), we subjected the animals to a high dose of urethane anesthesia and then perfused the hearts with 0.9% normal saline. The brain and heart were removed and post-fixed in 4% paraformaldehyde, then sectioned 5 μm thick. Sections were placed on slides then stained with hematoxylin and eosin (H&E). Infarct area (μm^2^) was calculated by subtracting the infarcted region from the non-infarcted area. The degree of cardiac inflammation was quantified (by means of particle analysis and presented as percentage of the total image area) using ImageJ software (ImageJ 1.51g, Wayne Rasband, National Institutes of Health, United States).

### Micro-CT Scan

Rats were injected with Omnipaque intra-arterially at 0.4 mL over a period of 15 s, at 48 h post-reperfusion. Brain CT was performed using Quantum FX UCT system for 5 min, and BBB disruption was detected for Omnipaque leakage. A total of 0.5 mL of 4% Evans blue in normal saline (1:24) was injected via tail vein. Evans blue extravasation was assessed by IVIS Lumina K (Series III).

### Blood Pressure Measurement

Invasive blood pressure (BP) was recorded at 12 h after the last dose of DHI, using ADInstruments PowerLab 8/30 connected to bridge Amp, ML 221 as described (Parasuraman and Raveendran, 2012). Briefly, Animals were anesthetized with 1.5 g/Kg intraperitoneal (i.p.) urethane which was proven to have minimal effect on autonomic and cardiovascular systems (Hara and Harris, 2002). Surgical manipulation was carried out on the animals and heparinized saline (100 IU/mL), was filled unto the transducer in a clean polyethylene catheter and cannulated to the left CCA. The BP was continuously recorded for 5 min as waveform curve. BP curves were detected, and the software calculated their mean.

### Electrocardiography and Hemodynamic Measurement

Using PowerLab hardware, Electrocardiograph (ECG)/heart rate was recorded for 5 min after a 30 min equilibration, and processed with Lab chart 7 Pro software version 7.3.8^1^. We calibrated the volume and pressure with the help of MVPS-Ultra system. We set the Tidal volume at 119–289 μL. We anesthetized the rats followed by dissection and exposition of the right carotid artery, and then a Millar-Tip (2F tip size) conductance catheter was introduced into the artery and advanced into the left ventricle (LV) through the aortic valve. As soon as steady-state hemodynamics was achieved, we recorded and processed the PV loops. Parallel conductance was determined individually in all the rats using a 15 to 30 μL bolus of 15% saline given through the right venous catheter. To compute for cardiac parameters, we processed the PV loop data with chart7 software. At the end of the experiment, we anesthetized the animals in other to obtain blood from the abdominal aorta (Naghshin et al., 2009). We evaluated the cardiac function by measuring the left ventricular developed pressure (LVDP, i.e., left ventricle end systolic pressure minus left ventricle end diastolic pressure), Cardiac output (CO), and Relaxation time constant (Tau) (Abraham and Mao, 2015).

#### Cardiac Arrhythmia Classification

Electrocardiograph was continually recorded for 5 min during the last day of DHI administration, 14^th^-day post-reperfusion. We used the Lambeth conventional guide for the classification of arrhythmias (Curtis et al., 2013) as follows: a prolonged QRS complex [premature ventricular beat (PVB)], from three or more consecutive PVBs [ventricular tachycardia (VT)], and the permanent lack of determinable QRS [from start of ventricular fibrillation (VF)]. We scored the severity of arrhythmia using the following criteria: score 0 = PVB < 50 beats; 1 = PVB from 50 to 450; 2 = PVB > 500 beats or one episode of reversible VT or VF; 3 = more than one episode of reversible VT or VF (less than a minute); 4 = 1–2 min of total combination duration time of VT or VF.

### Myocardial Infarction Model

Animals were anesthetized, after tracheal intubation, the ventilation was provided with a small animal mechanical ventilator (Shanghai Precision Scientific Instrument, Co. Ltd.; ALC-V8 CHINA). A mid-sternal thoracotomy was done to expose the heart via ligation of the left anterior descending coronary artery (LADCA). Sham operation was carried out in the same way as the model, but ligation was not placed. Animals with an ejection fraction of 35∼50% were used in the experiment after 24 h of LADCA ligation.

### Western Blotting Analysis

Proteins from the heart tissue were extracted with ice-cold lysis buffer and assayed using the BCA protein assay kit standardized to BSA. The membranes were incubated overnight at 4°C with specific primary antibodies against β_1_-AR, β_2_-AR, β_3_-AR, and Protein kinase C epsilon (PKCε). Blots were incubated in anti-rabbit and anti-mouse secondary antibody for 2 h. Proteins were detected using the enhanced chemiluminescence (ECL) system.

#### Enzyme-Linked Immunosorbent Assay

After the LV PV-loop measurement, rats were anesthetized to obtain the serum from the abdominal aorta, for the detection and quantification of the levels of Noradrenaline (NE) and Tyrosine hydroxylase (TH) in the serum by the ELISA method.

### Immunohistochemistry

Tissue sections were incubated overnight at the temperature of 4°C with Anti- β_1_-AR, Anti-β_2_-AR, or anti-β_3_-AR antibody at 1:300 1 μg/mL followed by incubation with secondary antibody for 1 h at 37°C. The immunoreactivity was detected using 3, 3′-diaminobenzidine tetrahydrochloride as the chromogen. The section was counterstained with hematoxylin. β-AR-positive staining was identified by the presence of brown staining of cells specifically present around the cytoplasm.

### Statistical Analysis

Data from all the experiments were presented as mean ± SEM. We computed the statistical differences using one-way analysis of variance followed by *post hoc* analysis with Dunnett’s *t*-test derived from the same estimate of the error variance. *p* < 0.05 is considered significant and *p* < 0.01 is considered highly significant.

## Results

### DHI Ameliorates Brain and Heart Infarcts at the Early Time Point of Reperfusion

**FIGURE 1:**
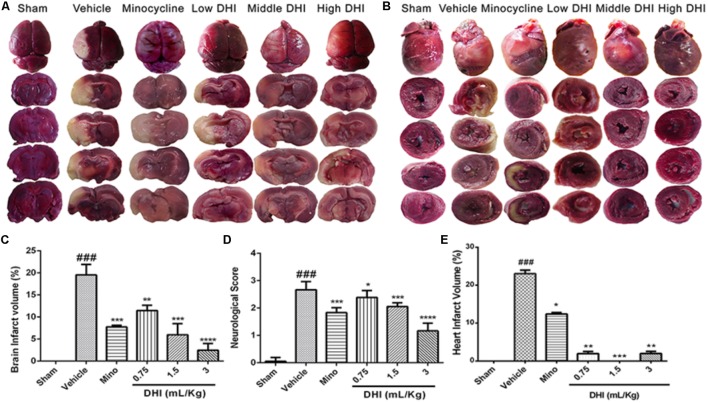
Danhong injection (DHI) attenuates brain infarct and neurological deficits at the early time point of reperfusion (*n* = 5 in each group). **(A)** Whole brain and brain slices stained with TTC 48 h after CI/RI. **(B)** Transverse sections of the hearts from the same animals stained with TTC 48 h post-reperfusion. Infarct was significantly detected in the vehicle (^###^*p* < 0.001 vs. sham) but decreased in the treatment groups (^∗^*p* < 0.05, ^∗∗^*p* < 0.01, and ^∗∗∗^*p* < 0.001 vs. vehicle; **B,E**). **(C)** MCAO procedure caused a marked increase in infarct volume (^###^*p* < 0.001 vs. sham) but decreased significantly in drug groups (^∗∗^*p* < 0.01, ^∗∗∗^*p* < 0.001, and ^∗∗∗∗^*p* < 0.0001 vs. vehicle). **(D)** Neurological deficit scores decreased statistically in drug-treated groups (^∗^*p* < 0.05, ^∗∗∗^*p* < 0.001, and ^∗∗∗∗^*p* < 0.0001 vs. vehicle) but remained high in the vehicle group (^###^*p* < 0.001 vs. sham).

TTC stain indicated a complete absence of infarct in the sham operated rats (**Figure 1A**). Brain and heart infarct increased significantly in the vehicle group (^###^*p* < 0.001 vs. sham). As expected, minocycline decreased the brain infarct volume (from 19.53 ± 2.38 to 7.75 ± 0.39%, ^∗∗∗^*p* < 0.001 vs. vehicle) and the heart infarct volume (from 23.03 ± 0.94 to 12.40 ± 0.39% vs. vehicle; ^∗^*p* < 0.05). Low, middle, and high doses of DHI also decreased the brain infarct volume (from 19.53 ± 2.38 to 11.47 ± 1.18%, 5.96 ± 2.52%, and 2.46 ± 1.52%; ^∗∗^*p* < 0.01) and the heart infarct volume from (from 23.03 ± 0.94 to 1.96 ± 0.56%, 0.0 ± 0.0%, and 1.99 ± 0.57%; ^∗∗^*p* < 0.01 and ^∗∗∗^*p* < 0.001 vs. vehicle), respectively (*n* = 6 in each group, **Figures 1A–C,E**).

### DHI Ameliorates Neurologic Deficit

Neurologic deficit score (NDS) was accessed by tail suspension and open field test. Rats induced with 60 min CI/RI displayed contralateral forelimb flexion with hindlimb extensor in the vehicle group (Supplementary Material Online Video). Sham-operated rats showed no neurologic deficit. However, there was significantly higher NDS in the vehicle group (###*p* < 0.0001 vs. sham). As a positive control, minocycline significantly decreased the NDS (from 2.67 ± 0.12 to 1.83 ± 0.07, ^∗∗∗^*p* < 0.0001 vs. vehicle *n* = 6). Comparatively, DHI at low, medium, and high doses also significantly decreased NDS (from 2.67 ± 0.12 to 2.39 ± 0.10, 2.06 ± 0.06, and 1.17 ± 0.11; ^∗^*p* < 0.05 and ^∗∗∗^*p* < 0.0001 vs. vehicle), respectively, with improved functional outcome (*n* = 6 in each group, **Figure 1D** and Supplementary Material Online Video).

### DHI Reduced Brain Infarct Further at a Late Time Point of Reperfusion and Rendered BBB Protection

**FIGURE 2:**
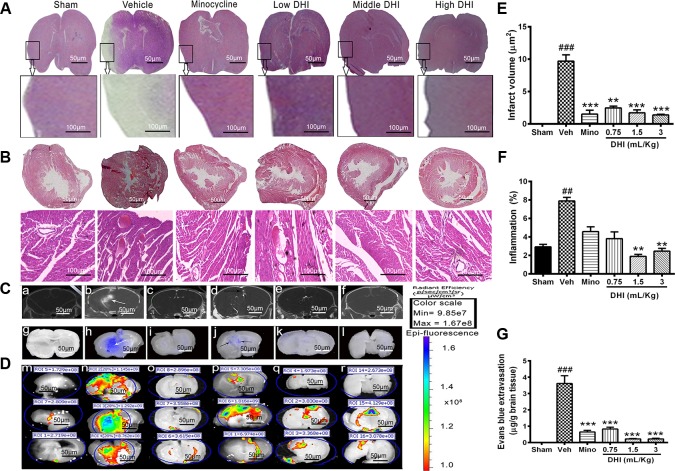
Danhong injection reduced brain infarct further and protected the BBB against damage caused by CI/RI. **(A,B)** Are brain and heart slices respectively, stained with H&E at the late time point of reperfusion (endpoint of the study, 14^th^ day of the experiment). Infarct disappeared completely within 14 days in the minocycline and DHI treated groups (^∗∗^*p* < 0.01 and ^∗∗∗^*p* < 0.001 vs. vehicle; **A,E**) but remains visible in the vehicle group (^###^*p* < 0.001 vs. sham; **A,E**). Histopathological examination of the hearts slices showed normal architecture in the sham and DHI groups. Sections from vehicle treatment reveal inflammation (^##^*p* < 0.001 vs. sham; **B,F**) but were ameliorated in in DHI middle and high group (^∗∗^*p* < 0.01 vs. vehicle; **B,F**). **(C)** CT-scan at 48 h after MCAO and intra-arterial injection of CT contrast agent (Omnipaque). (b) (White arrow) shows hyper-dense Micro-computed topographic (m-CT) lesions in the infarcted area, and Omnipaque leakage contrary to sham, minocycline, and doses of DHI (a,c,d–f). **(D)** Evans blue extravasation (g–l) and fluorescence image of Evans blue dye (m–r). **(G)** Quantitative bar chart of Evan blue dye in μg/g of brain tissue. Vehicle group showed Evans blue extravasation as a sign of BBB damage (^###^*p* < 0.001 vs. control; white arrows, h). No leakage in the sham group (g,m, with less fluorescence radiant efficacy, *p* > 0.05). Doses of DHI ameliorated BBB leakage in the same way as 20 mg/kg minocycline (^∗∗∗^*p* < 0.001 vs. vehicle; j–l,p–r). *n* = 3 in each group, data are presented as mean ± SEM.

We investigated if DHI reduced brain infarct further at a late time point of reperfusion. Tissue slices from the sham appeared normal after H&E staining. Those from the vehicle (model) group showed visible pathological changes in the brain including signs of inflammation in the heart (**Figures 2A,B,E,F**). Furthermore, minocycline decreased infarct volume further (from 9.70 ± 0.97 to 1.52 ± 0.60 μm^2^, ^∗∗∗^*p* < 0.0001 vs. vehicle) without affecting the heart (**Figures 2A,B,E**). Moreover, DHI treatment at low, middle, and high dose also decreased brain infarct volume further (from 9.70 ± 0.97 to 5.49 ± 1.80 μm^2^, 2.38 ± 1.13 μm^2^, and 1.39 ± 0.11 μm^2^; ^∗∗^*p* < 0.001 and ^∗∗∗^*p* < 0.0001 vs. vehicle), respectively (*n* = 3 in each group, **Figures 2A,E**). Middle and high doses but not the low doses of DHI, decreased myocardial inflammation (from 7.90 ± 0.38 to 1.90 ± 0.21%, and 2.46 ± 0.30% ^∗∗^*p* < 0.01 vs. vehicle), respectively. Statistically, minocycline did not reduce the inflammation compared to the vehicle group (*p* > 0.05). Inflammation was almost absent in the sham group (**Figures 2B,F** and Supplementary Figure 4).

Micro-CT imaging, Evans blue stain, and EPI-Fluorescence spectrometer were used to detect and quantify the BBB integrity (**Figures 2C,D,G**). No Omnipaque leakage and Evans blue extravasation in the sham group, and insignificant detectable spectra (**Figures 2Ca,g,Dm**), respectively. However, CI/RI caused a significant leakage of the BBB (^###^*p* < 0.0001 vs. sham, **Figures 2Cb,h,Dn,G**), respectively. As expected, treatment with minocycline significantly reduced Evans blue extravasation (from 4.1704 ± 0.658 to 0.652 ± 0.259 μg/g, ^∗∗∗^*p* < 0.0001 vs. vehicle; **Figures 2Ci,G**), as well as Omnipaque leakage, and the radiant efficacy of Epi-fluorescence spectra (**Figures 2Cc,Do**), respectively. Interestingly, treatment with DHI dose-dependently decreased Evans blue extravasation (from 4.170 ± 0.658 to 0.831 ± 0.299 μg/g; 0.227 ± 0.063 μg/g; and 0.176 ± 0.082 μg/g, ^∗∗∗^*p* < 0.0001 vs. vehicle; **Figures 2Cj–l,G**), as well as Omnipaque leakage (**Figure 2Cd–f**). Epi-Fluorescence image of Evans blue also confirmed this finding in three independent animals (**Figure 2Dp–r**, *n* = 3 in each group).

### DHI Decreased the Mortality Rate

Kaplan–Meier survival curve shows that survival proportion of the stroke-induced rats were significantly lower in the low and middle dose of DHI, especially during the acute period of reperfusion (first 72 h), compared to the vehicle (^∗^*p* < 0.05 vs. vehicle). No mortality was found with the sham within the14 days period of data collection (*n* = 7 in each group, Supplementary Figures 1A,B). High dose of DHI at the early stage of reperfusion (first 72 h) but not the late stage aggravated the death rate.

### DHI Ameliorates Cardiac Dysfunction

#### Effect on BP Levels

**FIGURE 3:**
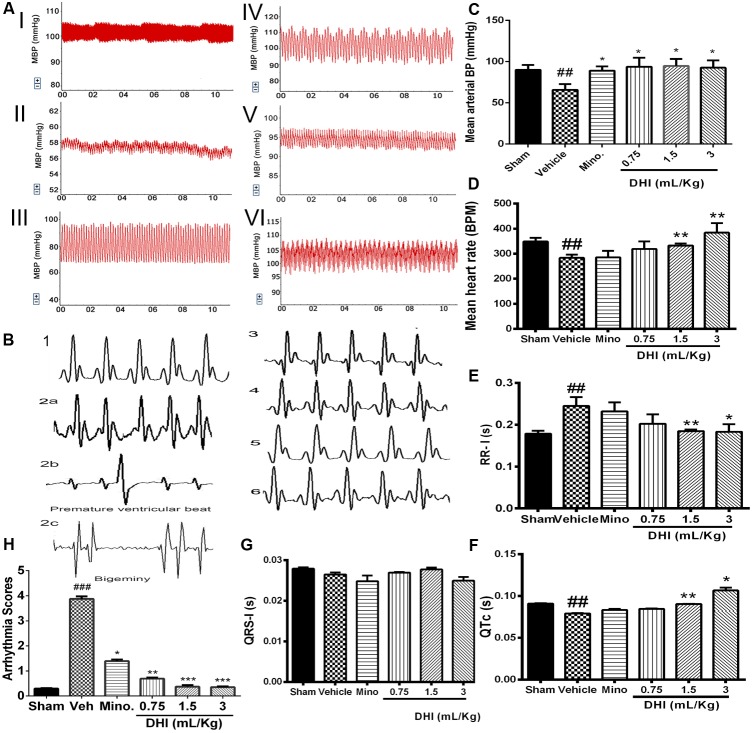
Danhong injection ameliorated CI/RI-induced cardiac dysfunction. **(A)** Representative traces of arterial BP: sham (I), vehicle (II), minocycline (III), and DHI: low (IV), middle (V), and high dose (VI), respectively. **(C)** Bar graph representation of **(A)**. CI/RI induced a low arterial BP in the vehicle (^##^*p* < 0.01 vs. sham). Doses of DHI restored normal BP (^∗^*p* < 0.05 vs. vehicle). Mean arterial BP was also normalized in the minocycline group (^∗^*p* < 0.05 vs. vehicle). **(D)** Bar graph representation of HR. CI/RI, induced abnormal low HR (∼270 bpm) in the vehicle compared to sham group (∼348 bpm; ^##^*p* < 0.01). DHI-middle and -high dose significantly normalized the HR (^∗∗^*p* < 0.01 vs. vehicle; **D**). HR remain unchanged in the low DHI and minocycline (20 mg/kg) group (*P* > 0.05 vs. vehicle). **(B)** ECG traces for sham (1), vehicle (2a), minocycline (3), and DHI groups (low, middle, and high doses, 4–6), respectively. **(E–G)** Bar graph representation of **(B)**. DHI significantly restored normal ECG RR-interval (^∗^*p* < 0.05, ^∗∗^*p* < 0.01 vs. vehicle; **E**) without affecting the QRS-I; **(G)**. The QTc duration decreased significantly in the vehicle group compared to the sham (^##^*p* < 0.01, **F**) but was brought to the baseline by the middle and high doses of DHI (^∗^*p* < 0.05, ^∗∗^*p* < 0.01 vs. vehicle; **F**). QTc duration in the minocycline and low DHI were not statistically different compared to the vehicle (*p* > 0.05; **F**). **(H)** Bar graph quantification of arrhythmia (2b,c). CI/RI induced cardiac arrhythmia in Wistar rats (score ^###^*p* < 0.001 vs. sham). There was a premature ventricular beat and bigeminy in the vehicle group (^###^*p* < 0.001 vs. sham; 2b,c) coupled with prolongation of the ECG RR-I waves (^###^*p* < 0.01 vs. sham; **E**) as signs of arrhythmia but were normalized by treatment with doses of DHI (^∗∗^*p* < 0.01, ^∗∗∗^*p* < 0.001 vs. vehicle; **H**) and minocycline (^∗^*p* < 0.05 vs. vehicle; **H**). *n* = 5 in each group, data are presented as mean ± SEM.

Cerebral ischemic/reperfusion injury caused arterial BP to fall below normal physiological range in the vehicle group (from 90.06 ± 5.88 to 65.79 ± 6.93 mm Hg, ^##^*p* ± 0.01 vs. sham; **Figures 3AI,II,C**). Mean BP of minocycline administered rats increased (from 65.79 ± 6.93 to 89.10 ± 5.13 mm Hg) **Figure 3AIII**. Also, treatment with doses of DHI (low, middle, and high) normalized the BP with increase (from 65.79 ± 6.93 to 95.85 ± 9.50 mm Hg, 94.82 ± 8.44 mm Hg, and 92.67 ± 8.94 mm Hg, ^∗^*p* < 0.05, *n* = 5 in each group; **Figures 3AIV–VI,C**).

#### Effect on ECG

Cardiac arrhythmia was scored based on the Lambeth convention guideline. VT = ventricular tachycardia and VF = ventricular fibrillation. The duration and incidence percentage of arrhythmia that resulted from CI/RI was significantly elevated in the vehicle compared to the sham and drug treatment groups (^###^*p* < 0.001 vs. sham; **Figures 3B2a–c,F**, Online Supplementary Figure 5 and Supplementary Table 3). Doses of DHI significantly protected against cardiac arrhythmia in the Wistar rats (^∗∗^*p* < 0.01 and ^∗∗∗^*p* < 0.001 vs. vehicle). Arrhythmia score (mean ± SEM, *n* = 5; **Figure 3H** and Supplementary Figure 5). The ECG RR-interval prolonged significantly in the vehicle group compared with sham (0.2447 ± 0.021 vs. 0.1785 ± 0.0072 s, ^###^*p* < 0.01 vs. sham, **Figure 3E**). Rats treated with minocycline were not significantly different from those of the vehicle in terms of RR-interval duration (0.232 ± 0.021 s, *p* > 0.05 vs. vehicle, **Figure 3E**). Impressively, treatment with middle and high doses of DHI showed cardiac regulation in terms of rhythms, with shortened RR-interval duration (from 0.2447 ± 0.021 to 0.185 ± 0.0039 s and 0.183 ± 0.018 s, ^∗∗^*p* < 0.01 and ^∗^*p* < 0.05 vs. vehicle, respectively; **Figure 3E**, *n* = 5 in each group). Decrease in duration of the trend of QTc was found in the vehicle group compared with sham (0.079 ± 0.0006 vs. 0.091 ± 0.00038 s, ^##^*p* < 0.01). Middle and high DHI increase the QTc duration to the baseline compared to those of the vehicle (0.090 ± 0.0002 s, 0.121 ± 0.0034 s vs. vehicle, ^∗∗^*p* < 0.01), respectively. Minocycline and low DHI are comparably the same with those of the vehicle (*p* > 0.05); **Figure 3F**, *n* = 5 in each group.

#### Effect on Heart Rate (HR)

Furthermore, left-brain infarction induced abnormal decrease in HR (from 348.82 ± 14.34 to 283.32 ± 13.04 bpm, ^###^*p* < 0.001 vs. sham) in the vehicle group. Similarly, HR remain unchanged in minocycline group compared to the vehicle (285.18 ± 26.19 vs. 283.32 ± 13.04 bpm, *p* > 0.05 vs. vehicle). Impressively, HR improved significantly in DHI middle and high doses with an increase (from 283.32 ± 13.04 to 332.66 ± 8.42 bpm and 384.06 ± 38.16 bpm, ^∗∗^*p* < 0.01 vs. vehicle; **Figure 3D**), respectively. No significant differences were detected between the drug group and the control (*p* > 0.05 vs. sham). However, low dose of DHI did not affect the reversal of the low HR (**Figure 3D**).

### Regulation of Hemodynamic Parameters and Neurotransmitter Activities of DHI

#### Left Ventricular Developed Pressure

**FIGURE 4:**
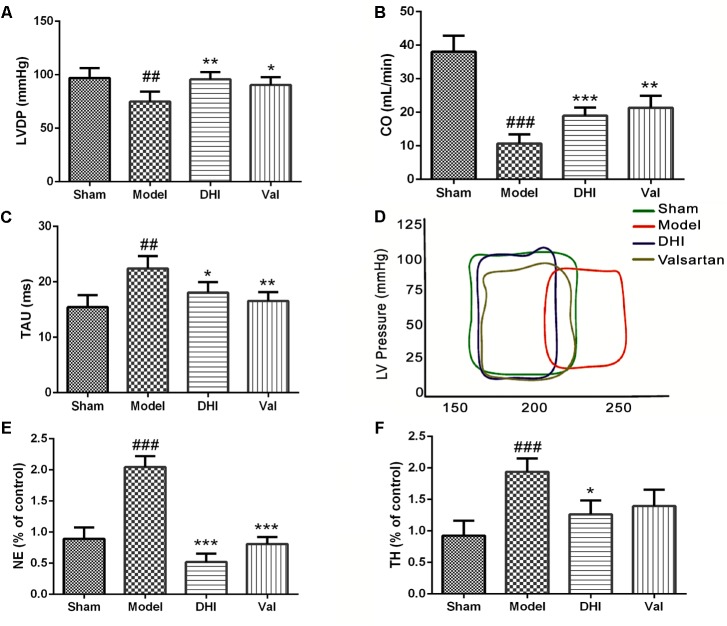
Danhong injection regulated the hemodynamic and neurotransmitter. Quantitative assessment of hemodynamics on cardiac function based on **(A)** left ventricular developed pressure (LVDP) = left ventricle-end systolic pressure minus left ventricle end diastolic pressure, **(B)** cardiac output (CO) and **(C)** relaxation time constant, were all significantly dysregulated in the MI rat model (^##^*p* < 0.01 and ^###^*p* < 0.001 vs. sham), but was regulated by treatment with DHI and valsartan (^∗^*p* < 0.05, ^∗∗^*p* < 0.01, and ^∗∗∗^*p* < 0.001 vs. model). **(D)** Left ventricular PV-loop. Valsartan and DHI-treatment enhanced improved ventricle and in the same way with the sham group contrarily to the model. **(E,F)** Bar graph representation of ELISA quantification for NE and TH in rat serum. The levels of NE and TH were increased in the model group compared to the sham (^###^*p* < 0.001 vs. sham). DHI significantly ameliorated the levels of NE and TH (^∗^*p* < 0.05 and ^∗∗∗^*p* < 0.001 vs. model), respectively (*n* = 6 in each group, data are presented as mean ± SEM).

The LV serves as the main pumping chamber of the heart. Failure to carry out the normal function may lead to hypoperfusion. Dysfunction of the LV may cause an abnormally high-pressure rise, and subsequently left atrial and pulmonary capillary pressure independent of systolic function (Kyhl et al., 2013). To find out if the cardioprotection by DHI in CI/RI model correlates with the neural signaling or cardiac local effect, we used MI model to measure and quantify the cardiac functionality after intramuscular DHI treatment (0.75 ml/kg/d). While normal LV pressure was maintained in the sham, MI significantly induced a downward pressure in the LV (^##^*p* < 0.01 vs. sham, **Figure 4A**). LVDP remained at the baseline for low dose of DHI and 10 mg/kg valsartan (^∗∗^*p* < 0.01; ^∗^*p* < 0.05 vs. model, **Figure 4A**).

#### Cardiac Output

The blood volume pumped through the heart per unit time known as CO, is produced by the heart (HR) to meet body’s demand for perfusion was maintained adequately in the sham-operated rats but significantly downregulated in the MI model (^##^*p* < 0.01) corresponding to the condition seen in the vehicle-treated group of CI/RI model. Valsartan treatment significantly (^∗^*p* < 0.05 vs. model) increased CO. Similarly, low dose of DHI also significantly (^∗^*p* < 0.05 vs. model) increased CO in MI-induced rats (**Figure 4B**).

#### Relaxation Time Constant (Tau)

The Tau was significantly elevated in the model group (^##^*p* < 0.01 vs. sham), however, it was reduced to baseline in valsartan treatment (*^∗∗^p* < 0.01 vs. model). Similarly, DHI also relaxed the Tau significantly (^∗^*p* < 0.05 vs. model). Comparably, DHI and valsartan treatment were statistically similar to the sham based on the Tau (**Figure 4C**).

#### Pressure-Volume Loop

Myocardial infarction induced a dysregulated Pressure vs. Volume loop at a defined line of contraction within the LV. On the contrary, treatment with valsartan or DHI kept the PV-loop within the maximum range of pressure developed by the ventricle at the corresponding LV volume in the same manner with the sham (**Figure 4D**).

#### Noradrenaline (NE)

Circulating level of neurotransmitter, NE was quantified by ELISA in DHI-treated MI-induced rats. NE was maintained at a normal physiological level in the sham-operated group. However, the levels of NE were significantly elevated in the model (^##^*p* < 0.01 vs. sham, **Figure 4E**). Treatment with valsartan and DHI reversed the low level of NE similar to those of the sham group (^∗∗^*p* < 0.01 vs. model, **Figure 4E**).

#### Tyrosine Hydroxylase (TH)

Similarly, TH, responsible for the catalysis of hydroxylation of L-tyrosine to I-DOPA as the rate-limiting step in the synthesis of catecholamine neurotransmitters dopamine, norepinephrine and epinephrine, was maintained at a normal physiological level in the sham-operated group. However, MI significantly elevated TH in the serum samples taken from the model (^##^*p* < 0.01 vs. sham). While the treatment with valsartan did not suppress TH levels statistically (*p* > 0.05 vs. model), DHI significantly suppressed TH levels (^∗^*p* < 0.05 vs. model, **Figure 4F**) indicating a better choice of therapy.

### The Regulation of β-Adrenergic Pathway of Brain–Heart Syndrome by DHI

#### Valsartan and DHI Ameliorates β-Adrenergic Accumulation Locally in the Heart

**FIGURE 5:**
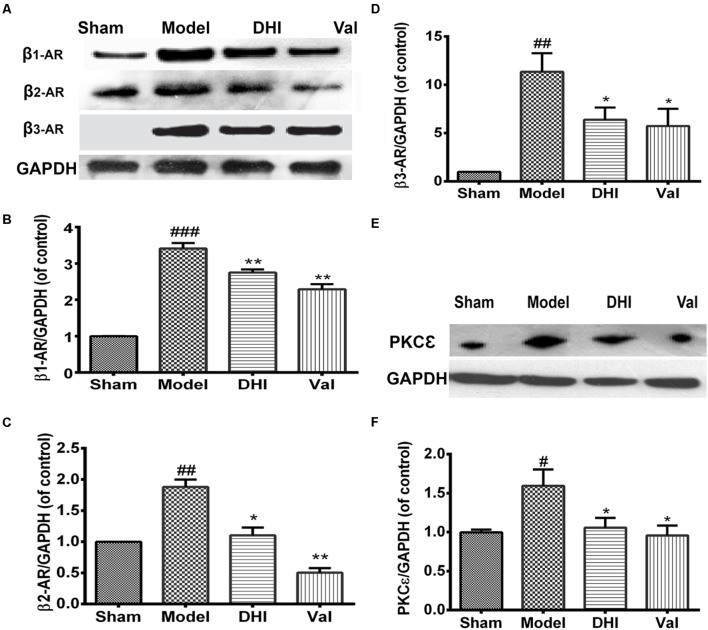
Similar and differential effects of DHI on β-AR subfamily member expression and downstream signaling proteins in MI model. **(A,E)** Are representative western blot bands of β-ARs and PKCε respectively in different groups. **(B–D,F)** Semi-quantitative analysis of heart’s β_1_-, β_2_-, and β_3_-AR, and PKCε in various groups. The over-expression of β_1_-AR, β_2_-AR, and β_3_-AR locally in the heart in the model group (^##^*p* < 0.01, ^###^*p* < 0.001 vs. sham) were significantly inhibited by DHI (^∗∗^*p* < 0.01 and ^∗^*p* < 0.05 vs. model; **A–D**). Also, PKCε over-expression in the heart (^#^*p* < 0.05 vs. sham), was significantly decreased by valsartan and DHI **(E,F)**, *n* = 3 in each group, data are presented as mean ± SEM.

Rats in the MI group displayed a significant increase in β_1_-adrenergic receptor expression compared to the sham group (^###^*p* < 0.001 vs. sham, **Figures 5A,B**). Valsartan and DHI also significantly downregulated β_1_-adrenergic expression (^∗∗^*p* < 0.01 vs. model). Similarly, β_2_-AR and β_3_-AR was at the baseline for sham and highly expressed in the model group (^##^*p* < 0.01 vs. sham, **Figures 5A,C,D**). Valsartan and DHI similarly downregulated β_2_- and β_3_-adrenergic expression in the MI model (^∗∗^*p* < 0.01 and ^∗^*p* < 0.01 vs. model, respectively, **Figures 5A,C,D**).

#### Valsartan and DHI-Treatment Regulates PKCε Expression in the Heart

We investigated the role played by PKCε protein in the MI model. There was an elevated PKCε level in the heart of the MI-induced rat model (^##^*p* < 0.01 vs. sham) but decreased by valsartan (^∗∗^*p* < 0.01 vs. model) as well as DHI (*^∗^p* < 0.01 vs. model, **Figures 5E,F**) which indicate that valsartan and DHI might be playing a regulatory role in PKCε pathway.

#### DHI-Treatment Ameliorated the β-AR Accumulation Remotely in Brain–Heart Syndrome

**FIGURE 6:**
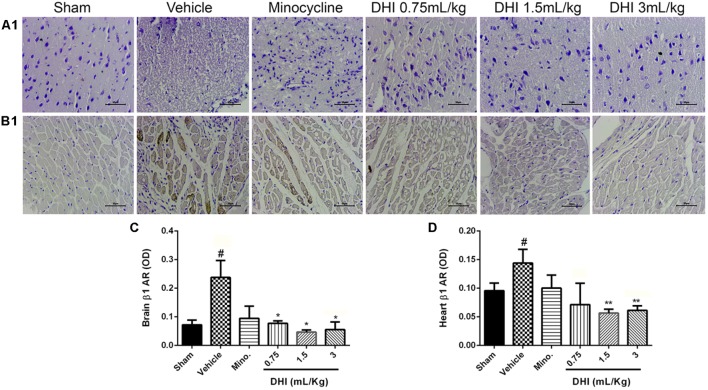
Similar and differential effects of DHI on β-AR subfamily member expression in CI/RI model. **(A_1_,B_1_)** Photomicrographs showing β_1_-AR expression in the brain and heart of Wistar rats, respectively. **(C,D)** Quantification of β_1_-AR in the brain and heart, respectively. Photomicrograph from IHC clearly showed brownish patches in the panels (**A_1_,B_1_** from the vehicle; ^#^*p* < 0.05 vs. vehicle) but almost invisible in both the sham and drug treatment groups (^∗^*p* < 0.05 and ^∗∗^*p* < 0.01 vs. vehicle). Minocycline treatment did not significant change β1-AR in the brain and heart (*p* > 0.05). All doses of DHI significantly ameliorated β_1_-AR accumulation in the brain (^∗^*p* < 0.05 vs. model; **A_1_,C**) where only DHI middle and high doses ameliorated the accumulation in the heart (^∗∗^*p* < 0.01 vs. model; **B_1_,D**). *n* = 3 in each group, image scale bar = 50 μm. Data are presented as mean ± SEM.

Immunohistochemistry (IHC) result showed significant expression of β_1_-AR in the brain and heart of rats induced with CI/RI (^#^*p* < 0.05 vs. sham, **Figures 6A–D**). β_1_-adrenergic expression in the brain and heart samples from rats treated with minocycline was not statistically different compared to those of the vehicle (*p* > 0.05 vs. vehicle, **Figures 6A–D**). All the three doses of DHI similarly decreased β_1_-AR expression in the brain (^∗^*p* < 0.05 vs. vehicle), whereas only the middle and high doses had the same effect in the heart (^∗∗^*p* < 0.01 vs. vehicle, **Figures 6A–D**).

**FIGURE 7:**
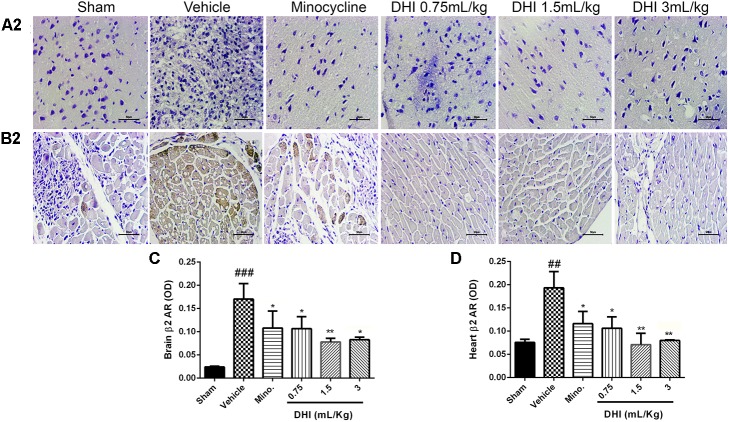
Danhong injection treatment adequately ameliorates β_2_-AR accumulation remotely in brain and heart of Wister rats. **(A_2_,B_2_)** Are photomicrographs of the brain and heart β_2_-AR assayed in IHC. **(C,D)** Quantification of β_2_-AR for brain and heart respectively. There are highly congested brownish patches in the brain **(A_2_)** and heart **(B_2_)** from the vehicle (^###^*p* < 0.001 and ^##^*p* < 0.01 vs. sham) but greatly reduced in drug treatment groups (^∗^*p* < 0.05 and ^∗∗^*p* < 0.01 vs. vehicle), respectively (*n* = 3 in each group, image scale bar = 50 μm). Data are presented as mean ± SEM.

Furthermore, β_2_-AR also expressed more in the brain and heart samples (^###^*p* < 0.001 and ^##^*p* < 0.01 vs. sham, **Figures 7A,B**), respectively, corresponding with the trend observed in the MI model. All doses of DHI suppressed β_2_-overexpression compared to those of vehicle (^∗^*p* < 0.05, ^∗∗^*p* < 0.01, **Figures 7A–D**). Minocycline significantly suppressed β_2_-overexpression in the brain and heart in a similar manner with those of DHI treatment (^∗^*p* < 0.05 vs. vehicle, **Figures 7A–D**).

Finally, the expression level of β_3_-AR in the brain and heart was modest in all the groups. No significant difference was found compared to the sham-operated rats and the vehicle (*p* > 0.05, Supplementary Figures 2A–D).

## Discussion

In this research work, we show that the multi-targeting DHI reversed cardiac abnormality via the β-adrenergic pathway, by suppressing the high level of the β-adrenergic expression in a rat model of brain–heart syndrome caused by CI/RI similar to MI and that the local β-AR signaling pathway is critical for cardiac protection after cerebral ischemic stroke. In a human stroke, poor prognosis is linked with pathologically low BP and accompanied by lack of cerebral perfusion leading to progressive deterioration of ischemic penumbra (Okumura et al., 2005). The severity of neurological deficits observed based on the localization and intensity of the brain infarction in our experiment play a role in cardiac dysfunction and mortality at the acute stage of stroke (Kallmünzer et al., 2012). However, reduced infarct volume as observed with DHI clearly reflected early recovery and improved functional outcome (Samuels, 2007; Guo et al., 2014).

Substance diffusion as a result of BBB disruption caused the Ominipaque penetration into the brain tissues but was inhibited by DHI treatment, suggesting that in ischemic conditions, Omnipaque penetration may occur more often, reflecting a frequent impairment of BBB. Moreover, BBB leakage coupled with local inflammatory responses and oxidative stress underline the mechanisms of neurochemical secretion into the general circulatory system, with a progressive increase in β-adrenergic accumulation and cardiac toxicity, causing detrimental effects on cardiac functions (Smalheiser, 2009; Thackeray et al., 2018).

The sequence in brain infarction accompanied a wild range of neurological disorders, suggesting that persistent, detrimental BBB disruption are associated with focal abnormal activity in areas associated with the infarct, as functional implications of such conditions are accompanied by neurological and cardiac dysregulation (Prosser et al., 2007) through series of pathological mechanisms. In addition, our results suggest that DHI played an important role in ameliorating brain infarction to prevent BBB disruption, an effect that could add to the therapeutic strategy of the multi-component medicine (Wang et al., 2014).

In addition, mortality rate caused by stroke incidence in human was estimated to every four deaths per minute with approximately 1 out of 20 deaths in the United States and ranked 4^th^ after heart disease among all causes of death (Benjamin et al., 2017). Clinical and experimental records indicate that reduced heart rate variability is a major predictor of mortality during the stroke (Norman et al., 2012). The mortality rate in a stroke model of rat encompasses many factors ranging from the animal strain, sex, age, methodology, and the drug being tested (Ström et al., 2013). In our study, high dose of DHI administered at the early time point of stroke (first 72 h), but not at the late time-point resulted in high mortality. This is interpreted to mean that, a great need exist in dose reduction during the critical time point of stroke as brain under severe pathophysiological stress might not handle certain quantities of drug irrespective of the efficacy of that drug.

Over-excitation of the neurotransmitter consequent to pathological conditions is implicated in cellular apoptosis (Lohse et al., 2003). Dopamine is involved in motor control, cognitive memory, and helps to support normal BP (Michael et al., 2008). The increased level of Th might be involved in the synthesis and accumulation of catecholamine neurotransmitters dopamine, norepinephrine (NE), and epinephrine (Zhu et al., 2002), and result in over-excitation of the excitatory neurons leading to toxicity including injury to the target organ. This harmful effect caused by the increased levels of Th and NE was also suppressed by DHI administration (**Figures 4E,F**), with improved LV pressure, Tau, and CO (**Figures 4B,C,E,F**).

Furthermore, the binding of β_2_-ARs to catecholamine (epinephrine and norepinephrine) has long been established to influence behavioral conditions and cardiac functions (Lohse et al., 2003). Neurological injury as a result of CI/RI following series of mechanisms cause β-ARs dysregulation through excessive circulating catecholamine (Vallon, 2011), and its release from myocardial nerve endings and influx of inflammatory cells (systemic) into the heart to induce myocyte toxicity and myocardial cell death (**Figures 4**–**7**). Our present findings show that neurologic impact of CI/RI on the heart caused the critical reduction of HR in correlation with arrhythmia and PV-loops which were all ameliorated by DHI (**Figures 3**, **4D**) (Laowattana et al., 2006; Doehner et al., 2018). In all, DHI showed a potent cardio-protection in terms of normalized BP and heart rhythm. However, similar effects observed with minocycline could be associated with its protection of brain to indirectly prevent cardiac dysfunction contrary to what was observed with multi-target agent DHI (Chen et al., 2016; Wei et al., 2016). Therefore, a more systematic study is required to show the effects of minocycline under physiological dysregulation.

While PKCε actvation has been linked with cardioprotection in setting of acute ischemic/reperfusion injury (Inagaki et al., 2006), activation of PKCε in chronic settings is detrimental and pharmacological inhibition of PKCε attenuated cardiac fibrosis and cardia dysfunction in a rat model of HF (Inagaki et al., 2008). A study shows the relationship between constitutive PKCε activation and a decrease in the base L-type Ca current density, and passivated the activation effect of the β-ARs on L-type Ca current (Yue et al., 2004). In a recent study by Hu et al. (2017) to show PKCε responses to cardiac overloads on myocardial sympathetic innervation, the authors found pressure overload significantly induced left ventricular dysfunction, increased plasma norepinephrine (NE), increase myocardial collagen deposit, upregulated the PKCε membrane-cytosol ratio, downregulated NE membrane fraction, increased tyrosine hydroxide (TH) nerve density with NE, and downregulated myocardial β_1_-AR mRNA expression (Hu et al., 2017). These findings are consistent with our present finding exception to the findings in β_1_-AR gene expression that was not confirmed. In view of current research on the role of PKCε in cardiac function and its pathway activation by the β-AR (Li et al., 2015), it is possible that PKCε might indirectly play an active role with the β-ARs signaling pathway during the brain–heart syndrome, however, this requires further investigation.

Cardiac arrhythmia was described as a problem with the rate or rhythm of the heart thereby causing an irregular heartbeat. Also, it has been shown that reperfusion-induced cardiac arrhythmias in cerebral ischemic stroke (Sun et al., 2014).

Electrocardiograph record is an important method to determine the cardiac condition in stroke patients. The QRS duration and RR-interval measured in our ECG recording are some of the important factors that explain cardiac performance with regard to internal or external stimuli and regulated by the sympathetic and parasympathetic tone (Doehner et al., 2018). Clinical evidence shows that 75% of stroke patients have ECG abnormality and LV hypertrophy (Selvetella et al., 2003). The long RR-interval correlates with low HR as observed by us. This could be a result of insular cortex damage as reported in a related study (Laowattana et al., 2006), and may be associated with a typical characteristic of a direct effect of sympathetic and parasympathetic hyperactivity as a result of the array of infarct spread around major important centers responsible for autonomic control of the heart (Samuels, 2007) (**Figures 1**, **3**). In our experiment, the QRS and ST measured in all the experiment groups are not statistically different compared to the control and model, but the RR-interval indicated a contrary result. However, the normalized BP, HR, and ECG RR-interval observed with DHI administration is presumably linked and could be a direct/indirect effect of DHI-led penumbra neurons survival and BBB protection (**Figures 1**, **3**), making it a potent pharmacologic active remedy.

It is well-known that a failing human heart involving the LV increases end-diastolic volume (EDV) using Laplace mechanism and decreases the pumping ability of the ventricles (Lorell and Carabello, 2000), referred to as a decrease in the left ventricular ejection fraction (LVEF). In view of MI, this, therefore, is interpreted to suggest that CI/RI-associated MI leads to increased stroke volume (SV), Left atrial dilatation (increase left atrial diameter), increase in cardiac muscle mass, enlargement of LV, a decline of systolic and/or diastolic function, and ultimately, induces HF (Chen et al., 2016). Recent reports suggest that a high mortality rate caused by HF during CI/RI is at least in part, the evidence of the insufficiently available pharmacotherapy needed to prevent myocardial remodeling processes that lead to HF (Rabkin and Moe, 2015). Thus, the scientific and medical approach is to prevent syndrome by identifying and discovering natural products that could complement the existing therapies.

The implication of brain challenge, malfunctioning, and its causal relationship of cardiac dysfunction in the post-cerebral ischemic stroke subject is generally called brain–heart syndrome. Brain–heart syndrome is usually associated with post-stroke patients as an indicator of systemic complication. A patient with a brain–heart syndrome, if not treated may become hypertensive, the kidney may fail, and may develop a recurrent stroke. Since the brain and heart connection is multi-faceted, it requires a multi-component medicine such as DHI which have the advantage of multi-targets in brain–heart syndrome.

Danhong injection (made from the extracts of Radix Salvia miltiorrhizae and Flos Carthami tinctorii) have the advantage of multi-targets in various disease network and pathways. Previous studies on the Bioactivity-integrated UPLC/Q-TOF-MS revealed that DHI played an important role in suppressing inflammatory responses through the NF-κB dependent pathway (Jiang et al., 2015) and anti-cardiac hypertrophic effects (Jiang and Lian, 2015), of which nine potential anti-inflammatory ingredients (danshensu, protocatechuic acid, protocatechuic aldehyde, caffeic acid, hydroxysafflor yellow A, safflor yellow A, salvianolic acid A, salvianolic acid B, and salvianolic acid C) were identified. A study on immunity and inflammation suggest that the signaling via β_2_-AR modulates NF-κB function which has physiological and clinical relevance (Kolmus et al., 2015). During CI/RI, neurotransmitters and inflammatory cytokines are released abnormally among others when neurochemicals bind to receptors, a neurohormonal mechanism in which the adrenergic nervous system plays an active role, causing hyperactivity/toxicity due to its accumulation of binding to ARs in the human ventricular myocardium resulting in HF.

Furthermore, a recently published report suggests that pre-B-cell leukemia transcription factor-1, along with six other transcription factors (TFs) are putative target TFs for DHI-mediated protection against cerebral ischemic stroke (Wei et al., 2016). DHI components identified with the TF factor to modulate the β-ARs are 6-hydrogxymethyllumazin, danshensu, 1-hydroxytaxinine A, danshenxinkun A, dihydrovalepotriate, hesperetic acid, lithospermate B, 3- β-hydroxymethylenetanshiquinone, and arachidic acid. Coffeic acid was identified as the modulator of neuronal acetylcholine. There is evidence that suggests that β-ARs cooperate with TFs through signal transducer and activator of transcription-3 (STAT3) to regulate cardiac function (Balligand, 2016). Oxidative stress can lead to cell injury and may contribute to various disease such as stroke, MI, diabetes, cancer, and major disorders. Compounds of DHI with radical-scavenging activity could reduce free radical production and ameliorate the disease progression. In addition, important components of DHI that may remotely link to β-ARs through its antioxidant, anticoagulation, antithrombotic, and antifibrinolytic activities (He et al., 2012) are danshensu, protocatechuic aldehyde salvianolic acid B, rosmarinic acid, and hydroxysafflor yellow A.

## Conclusion

In this research work, we show that DHI ameliorates cardiac abnormality via the β-adrenergic pathway in a rat brain–heart syndrome caused by CI/RI. We confirm the ameliorating effect of DHI in neurological deficits, brain infarction, BBB dysfunction, death rate during the early and late time point of reperfusion. We further demonstrate that CI/RI induce significant alterations in arterial BP, ECG, and HR, which are all reversed by the multi-targeting DHI. Similar to MI, the local β-AR signaling pathway is critical for cardiac protection in the CI/RI model. Moreover, brain β-AR signaling also remotely contributes the DHI protection in the heart. This study has therefore set a stage for a further investigation into the molecular mechanisms involved in DHI protection of brain–heart syndrome. Finally, our findings show that DHI acts on multiple pathophysiological pathways and regulates the β-adrenergic signaling.

## Author Contributions

JO and YZhu conceived the idea and wrote the manuscript. JO performed MCAO surgery, tissue staining, micro-CT scan, drug administration, Western blot analysis, and immunohistochemistry experiments. JY and LW performed myocardial infarction model, western blot, and immunohistochemistry. TZ performed micro-CT scan. MY participated in drug administration and animal weighing. JO, GF, YZ, and YZhu reviewed and edited the final manuscript.

## Conflict of Interest Statement

The authors declare that the research was conducted in the absence of any commercial or financial relationships that could be construed as a potential conflict of interest.

## Footnotes

1 www.ADinstruments.com
