# Supplementary material for: Danhong Injection Reversed Cardiac Abnormality in Brain–Heart Syndrome via Local and Remote β-Adrenergic Receptor Signaling
Source: Front Pharmacol. 2018 Jul 3;9:692. doi: 10.3389/fphar.2018.00692 (PMC6037833; doi:10.3389/fphar.2018.00692)
Supplement: Supplementary file 2 [file Presentation_1.pptx]

## Slide 1
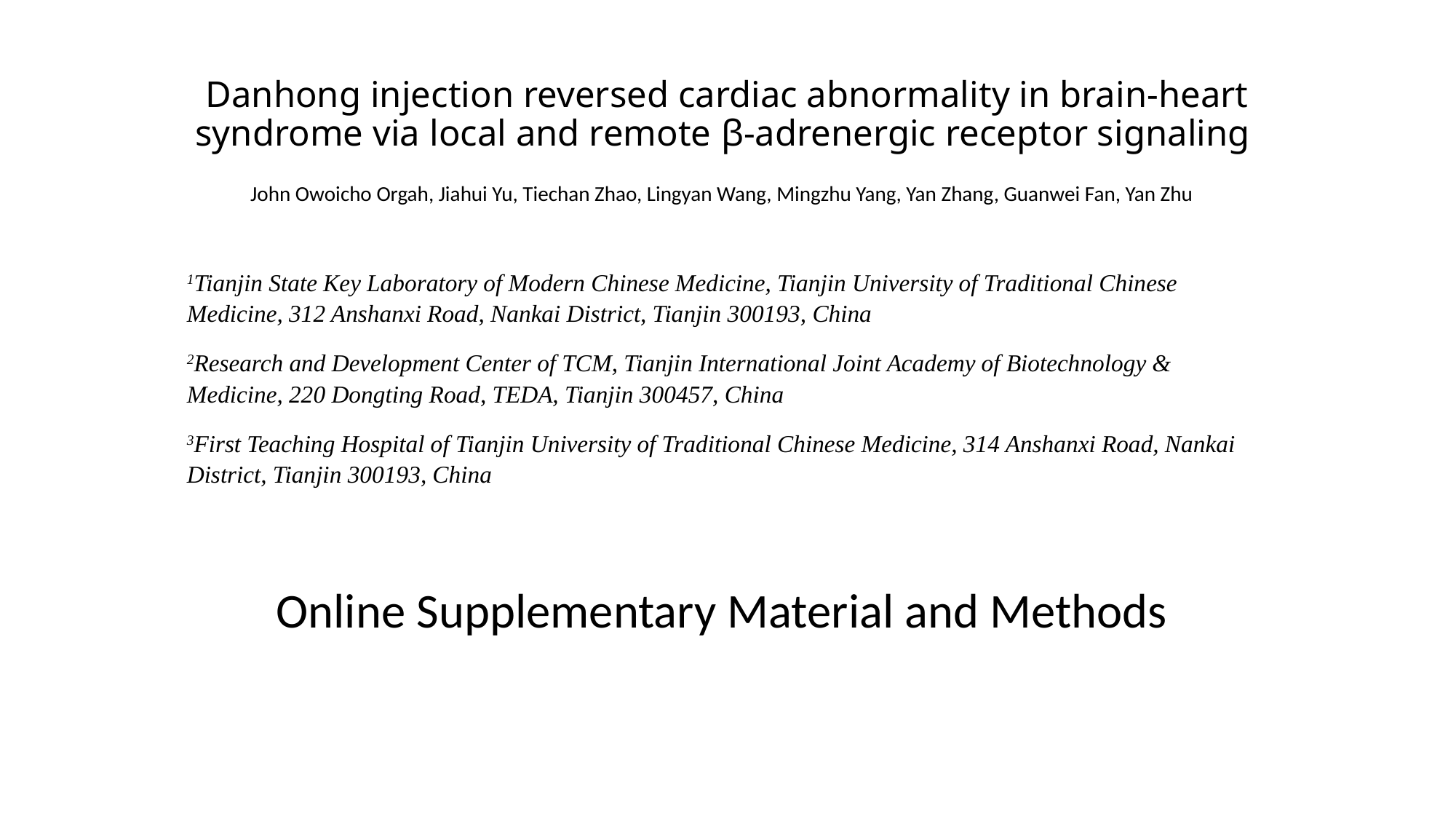

# Danhong injection reversed cardiac abnormality in brain-heart syndrome via local and remote β-adrenergic receptor signaling
John Owoicho Orgah, Jiahui Yu, Tiechan Zhao, Lingyan Wang, Mingzhu Yang, Yan Zhang, Guanwei Fan, Yan Zhu
1Tianjin State Key Laboratory of Modern Chinese Medicine, Tianjin University of Traditional Chinese Medicine, 312 Anshanxi Road, Nankai District, Tianjin 300193, China
2Research and Development Center of TCM, Tianjin International Joint Academy of Biotechnology & Medicine, 220 Dongting Road, TEDA, Tianjin 300457, China
3First Teaching Hospital of Tianjin University of Traditional Chinese Medicine, 314 Anshanxi Road, Nankai District, Tianjin 300193, China
Online Supplementary Material and Methods

## Slide 2
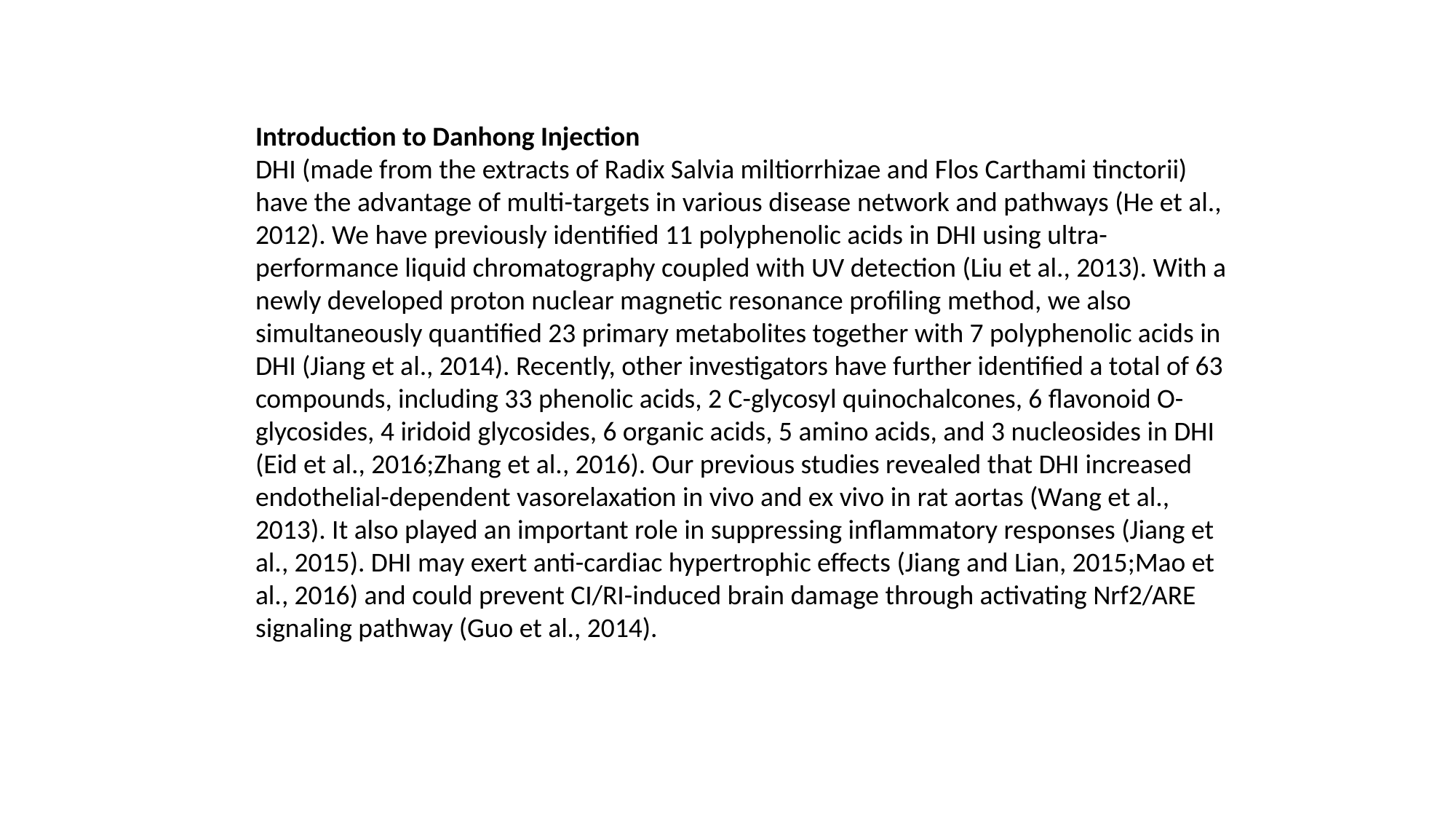

Introduction to Danhong Injection
DHI (made from the extracts of Radix Salvia miltiorrhizae and Flos Carthami tinctorii) have the advantage of multi-targets in various disease network and pathways (He et al., 2012). We have previously identified 11 polyphenolic acids in DHI using ultra-performance liquid chromatography coupled with UV detection (Liu et al., 2013). With a newly developed proton nuclear magnetic resonance profiling method, we also simultaneously quantified 23 primary metabolites together with 7 polyphenolic acids in DHI (Jiang et al., 2014). Recently, other investigators have further identified a total of 63 compounds, including 33 phenolic acids, 2 C-glycosyl quinochalcones, 6 flavonoid O-glycosides, 4 iridoid glycosides, 6 organic acids, 5 amino acids, and 3 nucleosides in DHI (Eid et al., 2016;Zhang et al., 2016). Our previous studies revealed that DHI increased endothelial-dependent vasorelaxation in vivo and ex vivo in rat aortas (Wang et al., 2013). It also played an important role in suppressing inflammatory responses (Jiang et al., 2015). DHI may exert anti-cardiac hypertrophic effects (Jiang and Lian, 2015;Mao et al., 2016) and could prevent CI/RI-induced brain damage through activating Nrf2/ARE signaling pathway (Guo et al., 2014).

## Slide 3
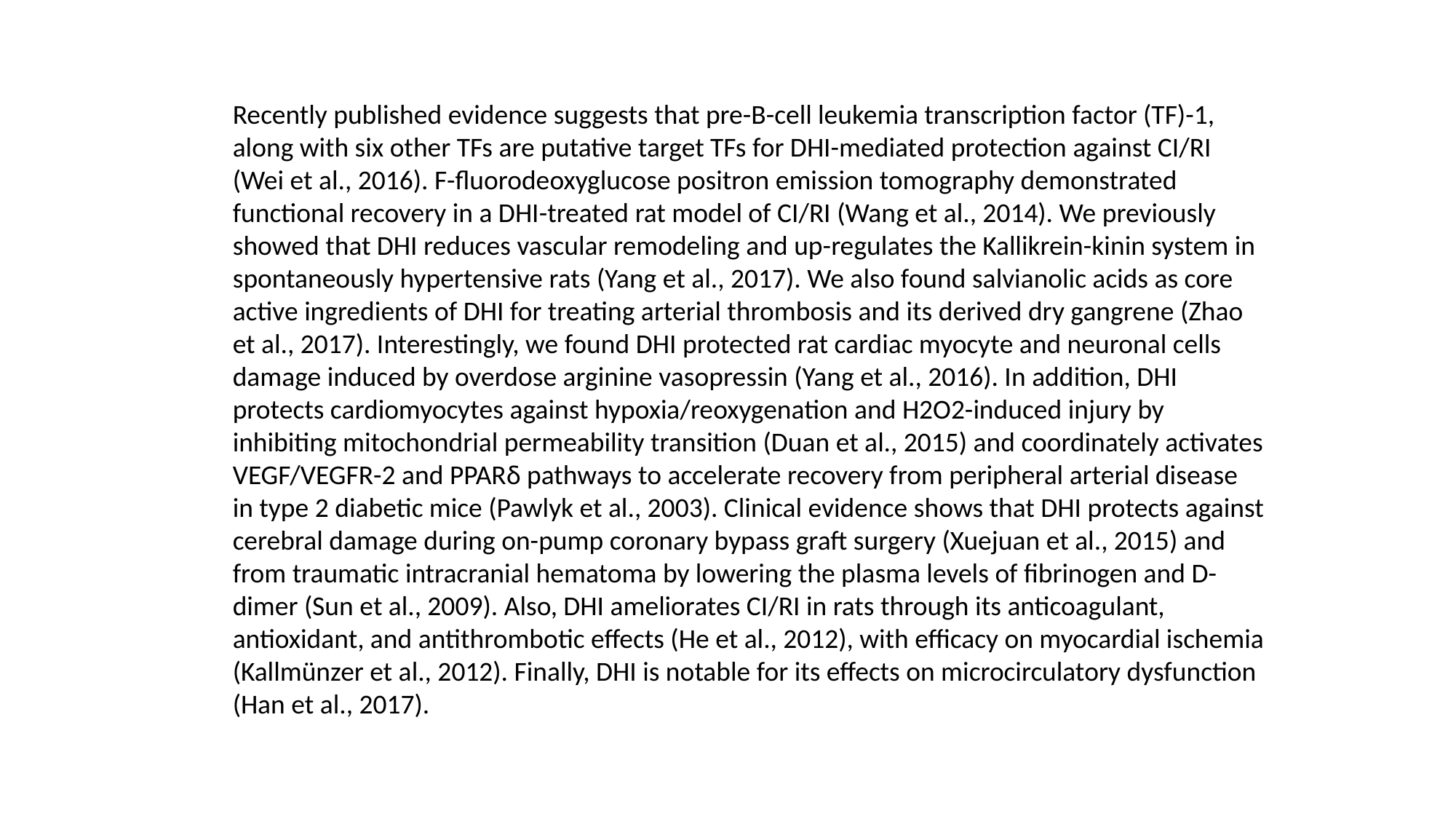

Recently published evidence suggests that pre-B-cell leukemia transcription factor (TF)-1, along with six other TFs are putative target TFs for DHI-mediated protection against CI/RI (Wei et al., 2016). F-fluorodeoxyglucose positron emission tomography demonstrated functional recovery in a DHI-treated rat model of CI/RI (Wang et al., 2014). We previously showed that DHI reduces vascular remodeling and up-regulates the Kallikrein-kinin system in spontaneously hypertensive rats (Yang et al., 2017). We also found salvianolic acids as core active ingredients of DHI for treating arterial thrombosis and its derived dry gangrene (Zhao et al., 2017). Interestingly, we found DHI protected rat cardiac myocyte and neuronal cells damage induced by overdose arginine vasopressin (Yang et al., 2016). In addition, DHI protects cardiomyocytes against hypoxia/reoxygenation and H2O2-induced injury by inhibiting mitochondrial permeability transition (Duan et al., 2015) and coordinately activates VEGF/VEGFR-2 and PPARδ pathways to accelerate recovery from peripheral arterial disease in type 2 diabetic mice (Pawlyk et al., 2003). Clinical evidence shows that DHI protects against cerebral damage during on-pump coronary bypass graft surgery (Xuejuan et al., 2015) and from traumatic intracranial hematoma by lowering the plasma levels of fibrinogen and D-dimer (Sun et al., 2009). Also, DHI ameliorates CI/RI in rats through its anticoagulant, antioxidant, and antithrombotic effects (He et al., 2012), with efficacy on myocardial ischemia (Kallmünzer et al., 2012). Finally, DHI is notable for its effects on microcirculatory dysfunction (Han et al., 2017).

## Slide 4
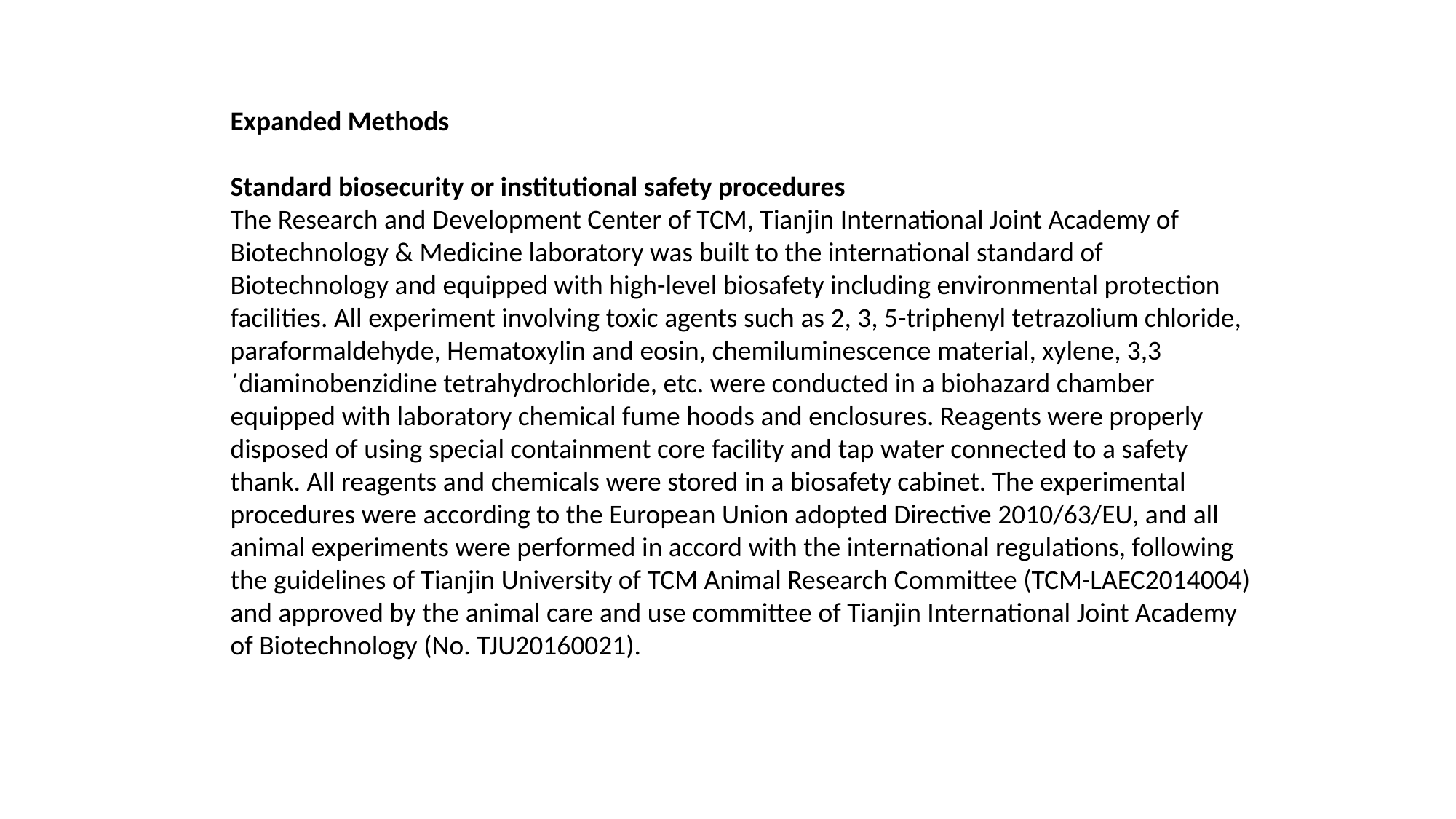

Expanded Methods
Standard biosecurity or institutional safety procedures
The Research and Development Center of TCM, Tianjin International Joint Academy of Biotechnology & Medicine laboratory was built to the international standard of Biotechnology and equipped with high-level biosafety including environmental protection facilities. All experiment involving toxic agents such as 2, 3, 5-triphenyl tetrazolium chloride, paraformaldehyde, Hematoxylin and eosin, chemiluminescence material, xylene, 3,3´diaminobenzidine tetrahydrochloride, etc. were conducted in a biohazard chamber equipped with laboratory chemical fume hoods and enclosures. Reagents were properly disposed of using special containment core facility and tap water connected to a safety thank. All reagents and chemicals were stored in a biosafety cabinet. The experimental procedures were according to the European Union adopted Directive 2010/63/EU, and all animal experiments were performed in accord with the international regulations, following the guidelines of Tianjin University of TCM Animal Research Committee (TCM-LAEC2014004) and approved by the animal care and use committee of Tianjin International Joint Academy of Biotechnology (No. TJU20160021).

## Slide 5
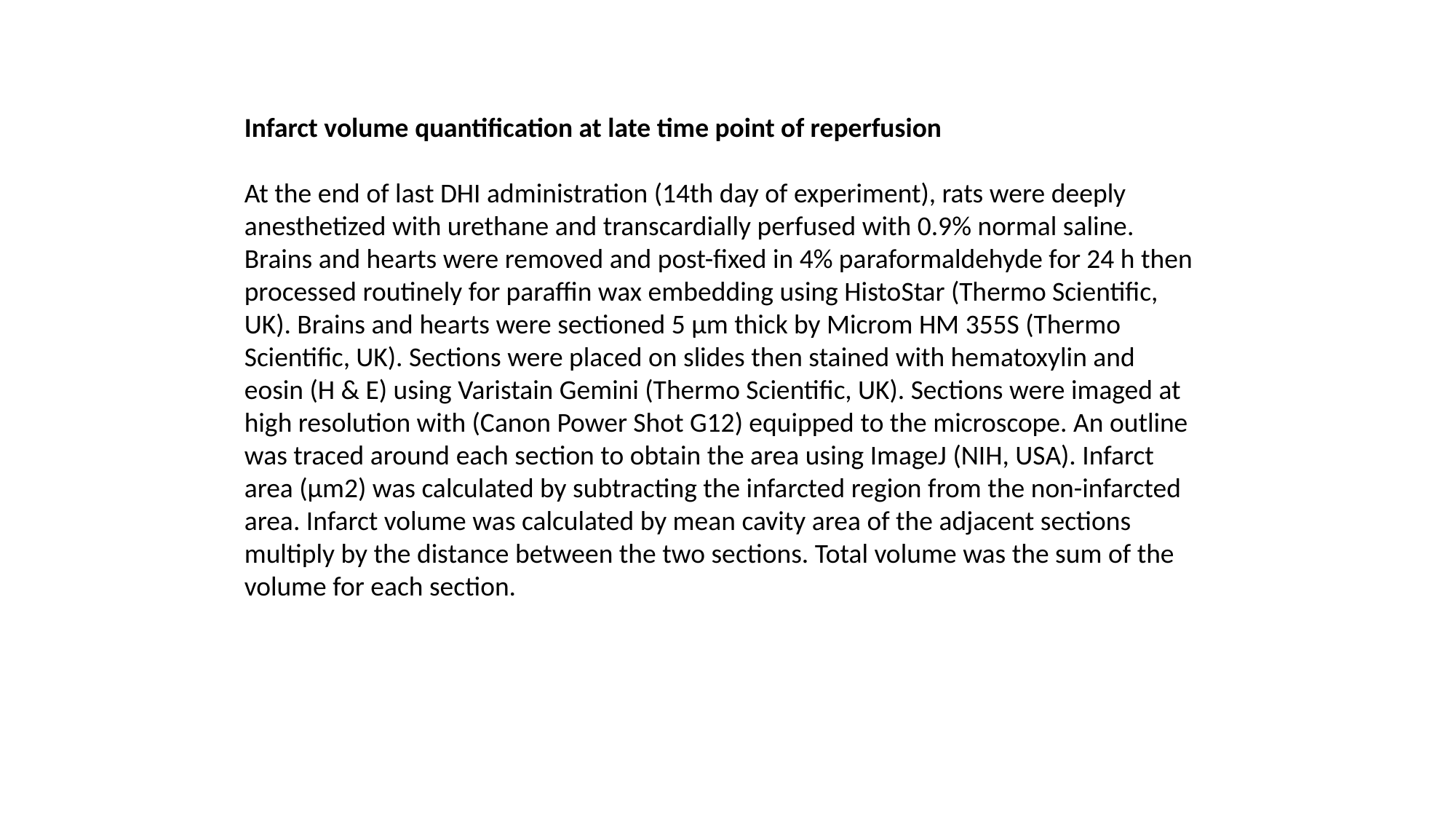

Infarct volume quantification at late time point of reperfusion
At the end of last DHI administration (14th day of experiment), rats were deeply anesthetized with urethane and transcardially perfused with 0.9% normal saline. Brains and hearts were removed and post-fixed in 4% paraformaldehyde for 24 h then processed routinely for paraffin wax embedding using HistoStar (Thermo Scientific, UK). Brains and hearts were sectioned 5 µm thick by Microm HM 355S (Thermo Scientific, UK). Sections were placed on slides then stained with hematoxylin and eosin (H & E) using Varistain Gemini (Thermo Scientific, UK). Sections were imaged at high resolution with (Canon Power Shot G12) equipped to the microscope. An outline was traced around each section to obtain the area using ImageJ (NIH, USA). Infarct area (µm2) was calculated by subtracting the infarcted region from the non-infarcted area. Infarct volume was calculated by mean cavity area of the adjacent sections multiply by the distance between the two sections. Total volume was the sum of the volume for each section.

## Slide 6
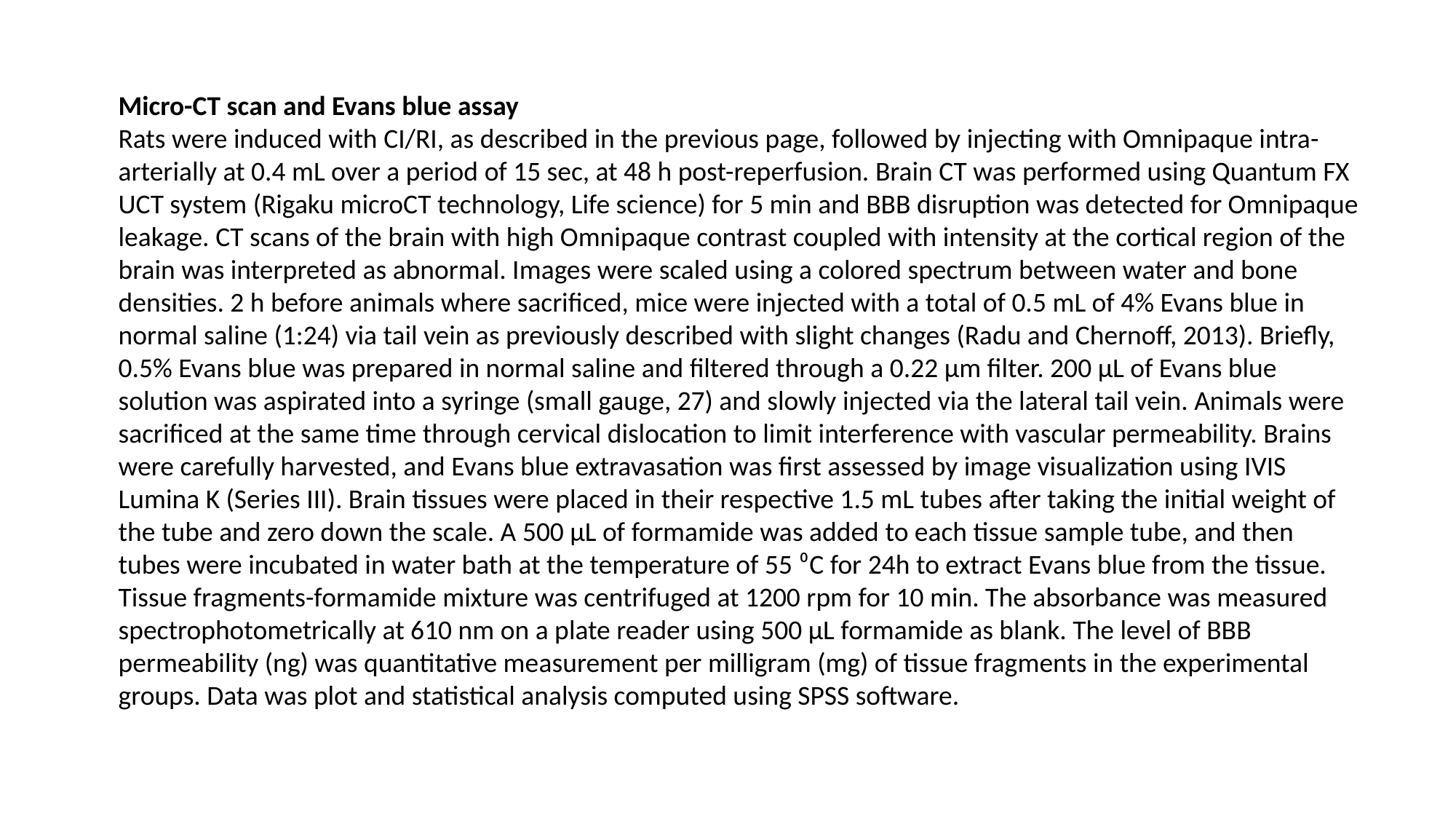

Micro-CT scan and Evans blue assay
Rats were induced with CI/RI, as described in the previous page, followed by injecting with Omnipaque intra-arterially at 0.4 mL over a period of 15 sec, at 48 h post-reperfusion. Brain CT was performed using Quantum FX UCT system (Rigaku microCT technology, Life science) for 5 min and BBB disruption was detected for Omnipaque leakage. CT scans of the brain with high Omnipaque contrast coupled with intensity at the cortical region of the brain was interpreted as abnormal. Images were scaled using a colored spectrum between water and bone densities. 2 h before animals where sacrificed, mice were injected with a total of 0.5 mL of 4% Evans blue in normal saline (1:24) via tail vein as previously described with slight changes (Radu and Chernoff, 2013). Briefly, 0.5% Evans blue was prepared in normal saline and filtered through a 0.22 µm filter. 200 µL of Evans blue solution was aspirated into a syringe (small gauge, 27) and slowly injected via the lateral tail vein. Animals were sacrificed at the same time through cervical dislocation to limit interference with vascular permeability. Brains were carefully harvested, and Evans blue extravasation was first assessed by image visualization using IVIS Lumina K (Series III). Brain tissues were placed in their respective 1.5 mL tubes after taking the initial weight of the tube and zero down the scale. A 500 µL of formamide was added to each tissue sample tube, and then tubes were incubated in water bath at the temperature of 55 ⁰C for 24h to extract Evans blue from the tissue. Tissue fragments-formamide mixture was centrifuged at 1200 rpm for 10 min. The absorbance was measured spectrophotometrically at 610 nm on a plate reader using 500 µL formamide as blank. The level of BBB permeability (ng) was quantitative measurement per milligram (mg) of tissue fragments in the experimental groups. Data was plot and statistical analysis computed using SPSS software.

## Slide 7
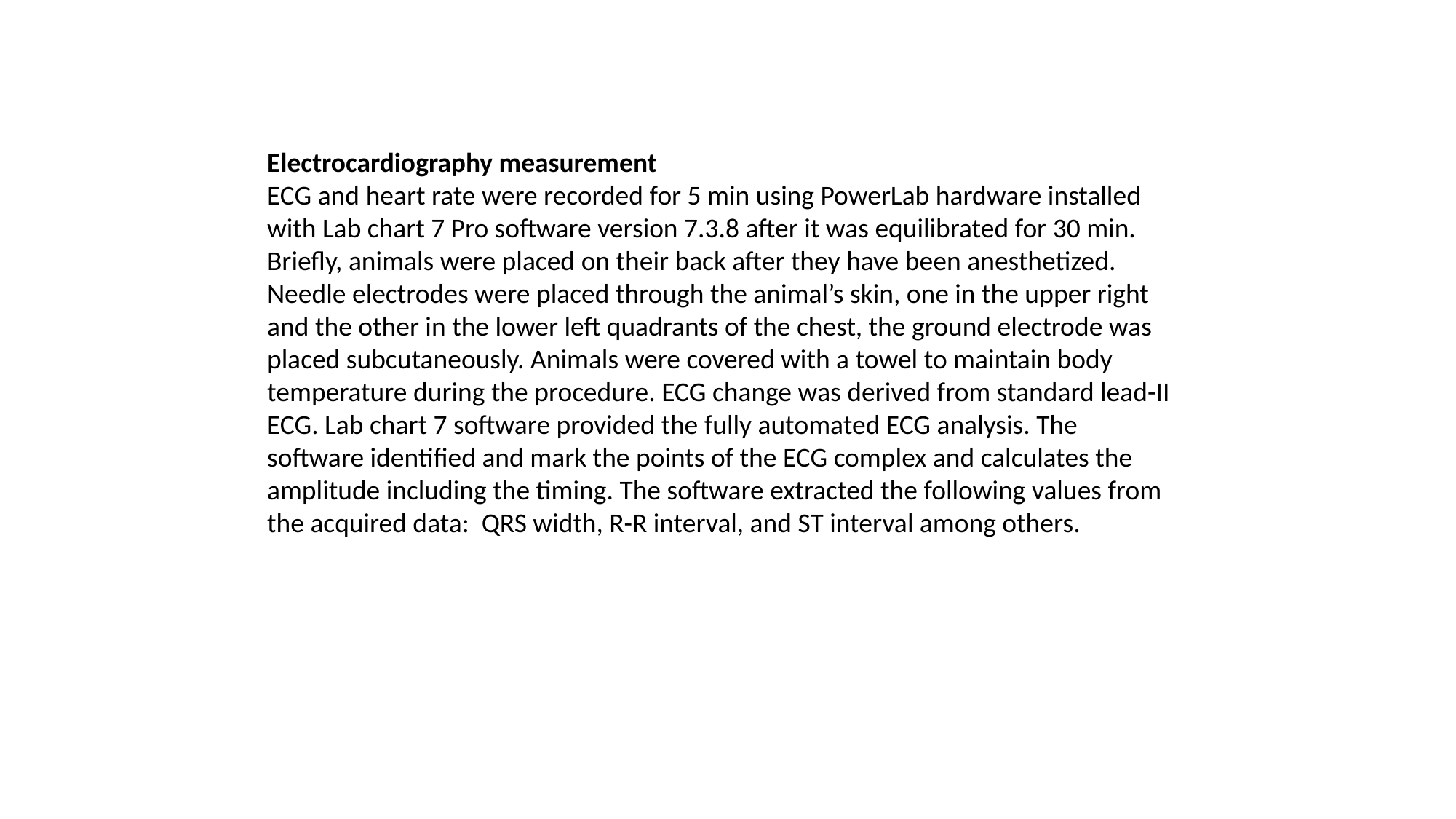

Electrocardiography measurement
ECG and heart rate were recorded for 5 min using PowerLab hardware installed with Lab chart 7 Pro software version 7.3.8 after it was equilibrated for 30 min. Briefly, animals were placed on their back after they have been anesthetized. Needle electrodes were placed through the animal’s skin, one in the upper right and the other in the lower left quadrants of the chest, the ground electrode was placed subcutaneously. Animals were covered with a towel to maintain body temperature during the procedure. ECG change was derived from standard lead-II ECG. Lab chart 7 software provided the fully automated ECG analysis. The software identified and mark the points of the ECG complex and calculates the amplitude including the timing. The software extracted the following values from the acquired data: QRS width, R-R interval, and ST interval among others.

## Slide 8
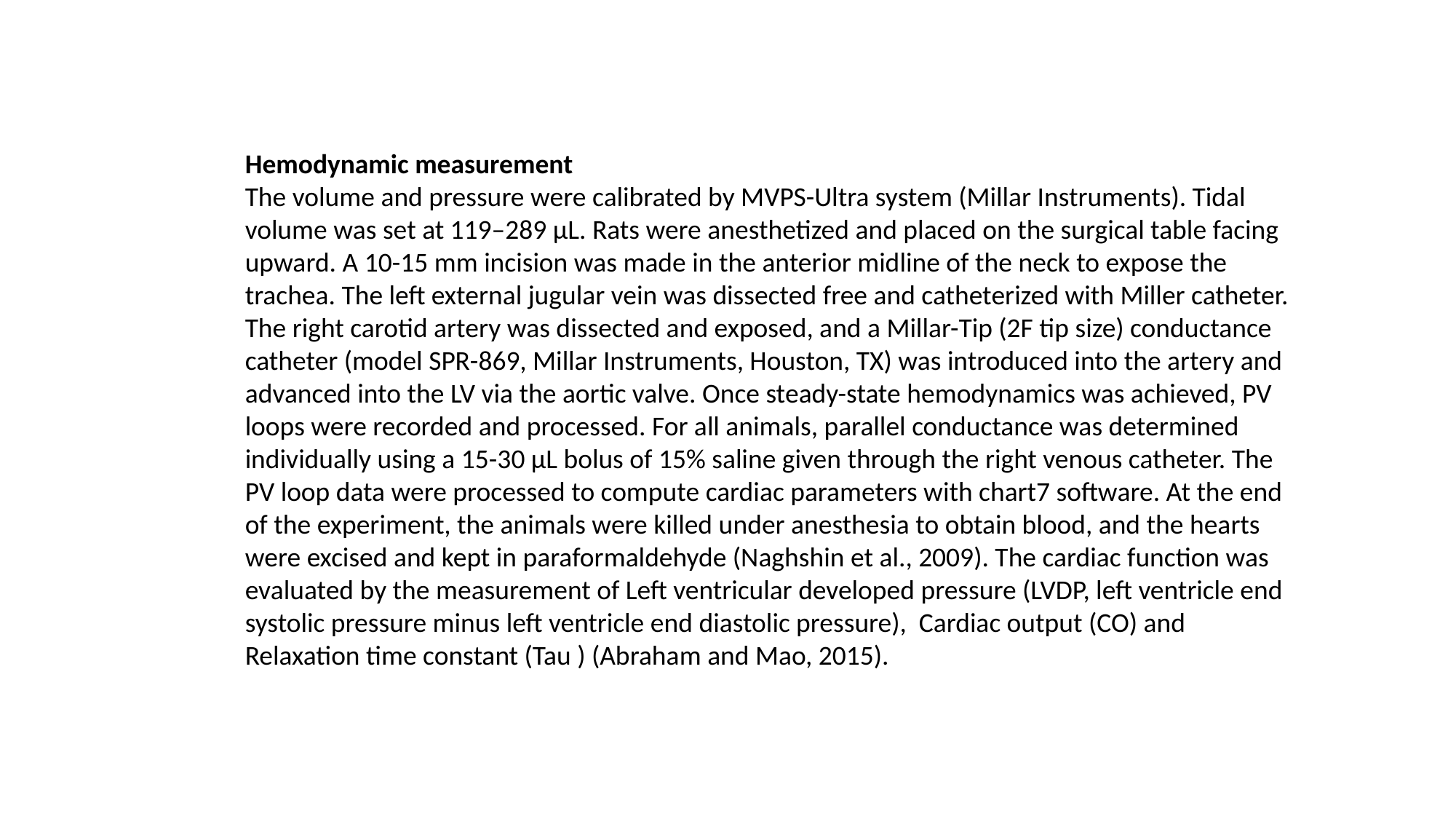

Hemodynamic measurement
The volume and pressure were calibrated by MVPS-Ultra system (Millar Instruments). Tidal volume was set at 119–289 μL. Rats were anesthetized and placed on the surgical table facing upward. A 10-15 mm incision was made in the anterior midline of the neck to expose the trachea. The left external jugular vein was dissected free and catheterized with Miller catheter. The right carotid artery was dissected and exposed, and a Millar-Tip (2F tip size) conductance catheter (model SPR-869, Millar Instruments, Houston, TX) was introduced into the artery and advanced into the LV via the aortic valve. Once steady-state hemodynamics was achieved, PV loops were recorded and processed. For all animals, parallel conductance was determined individually using a 15-30 μL bolus of 15% saline given through the right venous catheter. The PV loop data were processed to compute cardiac parameters with chart7 software. At the end of the experiment, the animals were killed under anesthesia to obtain blood, and the hearts were excised and kept in paraformaldehyde (Naghshin et al., 2009). The cardiac function was evaluated by the measurement of Left ventricular developed pressure (LVDP, left ventricle end systolic pressure minus left ventricle end diastolic pressure), Cardiac output (CO) and Relaxation time constant (Tau ) (Abraham and Mao, 2015).

## Slide 9
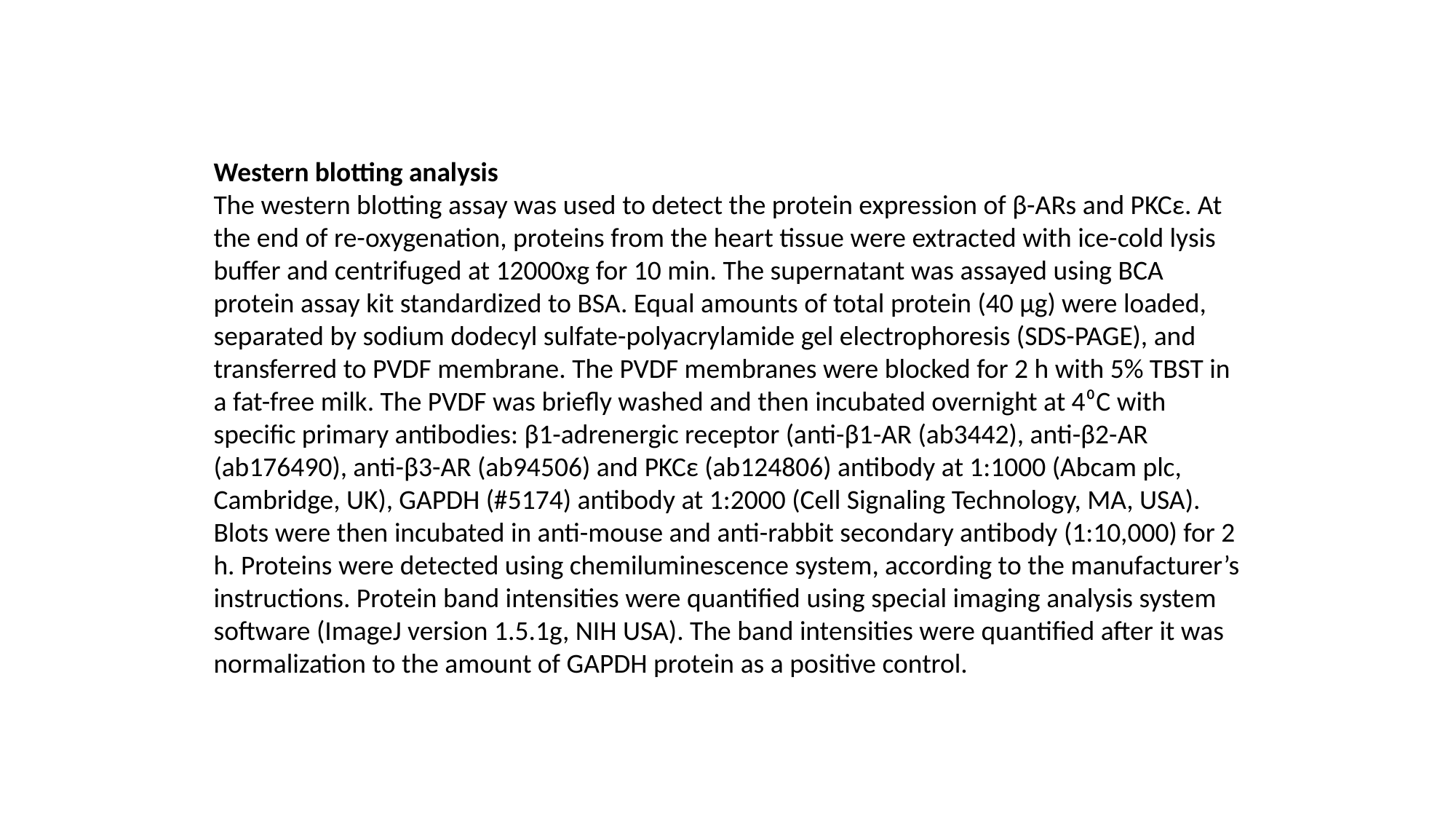

Western blotting analysis
The western blotting assay was used to detect the protein expression of β-ARs and PKCε. At the end of re-oxygenation, proteins from the heart tissue were extracted with ice-cold lysis buffer and centrifuged at 12000xg for 10 min. The supernatant was assayed using BCA protein assay kit standardized to BSA. Equal amounts of total protein (40 µg) were loaded, separated by sodium dodecyl sulfate-polyacrylamide gel electrophoresis (SDS-PAGE), and transferred to PVDF membrane. The PVDF membranes were blocked for 2 h with 5% TBST in a fat-free milk. The PVDF was briefly washed and then incubated overnight at 4⁰C with specific primary antibodies: β1-adrenergic receptor (anti-β1-AR (ab3442), anti-β2-AR (ab176490), anti-β3-AR (ab94506) and PKCε (ab124806) antibody at 1:1000 (Abcam plc, Cambridge, UK), GAPDH (#5174) antibody at 1:2000 (Cell Signaling Technology, MA, USA). Blots were then incubated in anti-mouse and anti-rabbit secondary antibody (1:10,000) for 2 h. Proteins were detected using chemiluminescence system, according to the manufacturer’s instructions. Protein band intensities were quantified using special imaging analysis system software (ImageJ version 1.5.1g, NIH USA). The band intensities were quantified after it was normalization to the amount of GAPDH protein as a positive control.

## Slide 10
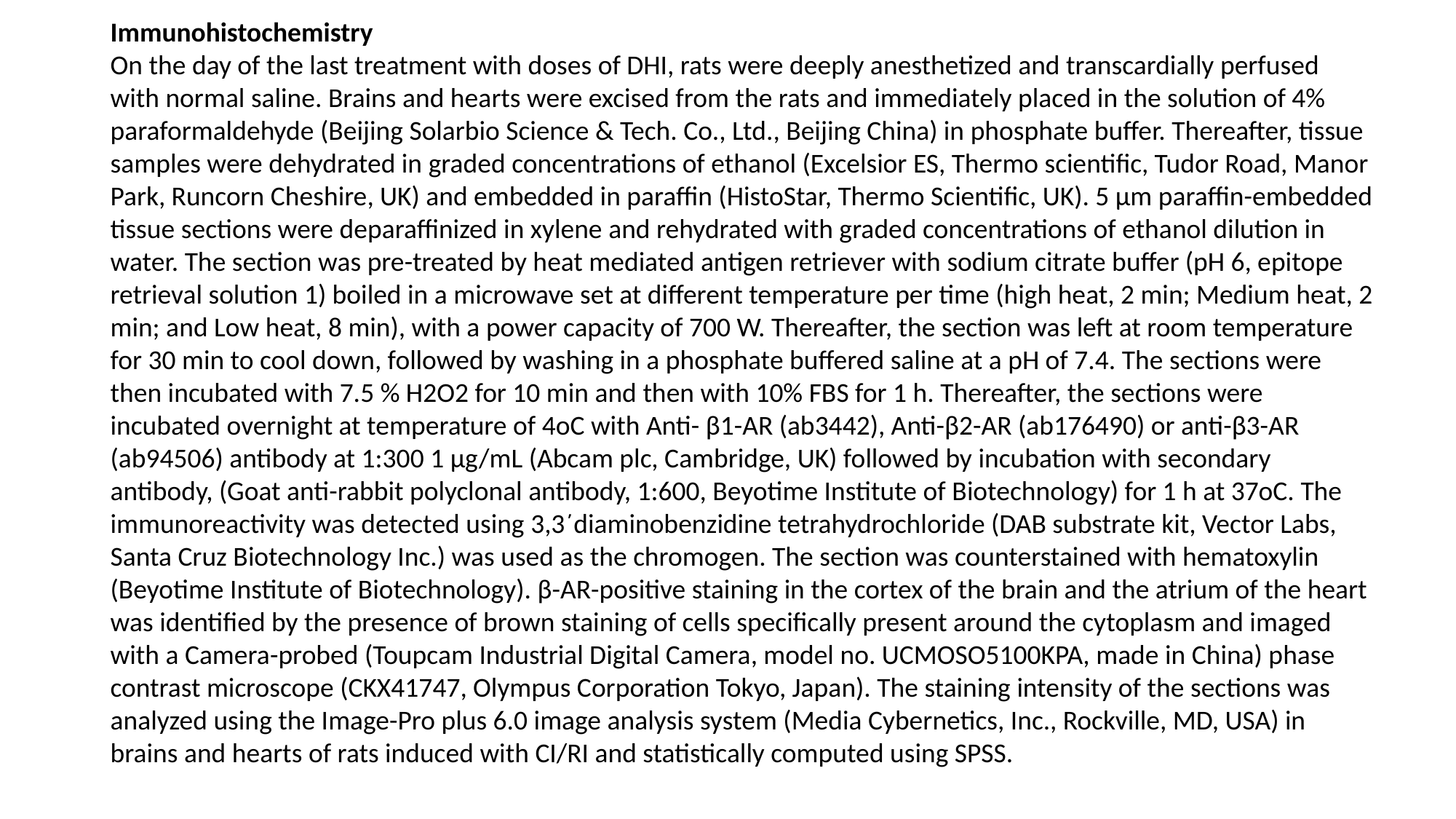

Immunohistochemistry
On the day of the last treatment with doses of DHI, rats were deeply anesthetized and transcardially perfused with normal saline. Brains and hearts were excised from the rats and immediately placed in the solution of 4% paraformaldehyde (Beijing Solarbio Science & Tech. Co., Ltd., Beijing China) in phosphate buffer. Thereafter, tissue samples were dehydrated in graded concentrations of ethanol (Excelsior ES, Thermo scientific, Tudor Road, Manor Park, Runcorn Cheshire, UK) and embedded in paraffin (HistoStar, Thermo Scientific, UK). 5 µm paraffin-embedded tissue sections were deparaffinized in xylene and rehydrated with graded concentrations of ethanol dilution in water. The section was pre-treated by heat mediated antigen retriever with sodium citrate buffer (pH 6, epitope retrieval solution 1) boiled in a microwave set at different temperature per time (high heat, 2 min; Medium heat, 2 min; and Low heat, 8 min), with a power capacity of 700 W. Thereafter, the section was left at room temperature for 30 min to cool down, followed by washing in a phosphate buffered saline at a pH of 7.4. The sections were then incubated with 7.5 % H2O2 for 10 min and then with 10% FBS for 1 h. Thereafter, the sections were incubated overnight at temperature of 4oC with Anti- β1-AR (ab3442), Anti-β2-AR (ab176490) or anti-β3-AR (ab94506) antibody at 1:300 1 µg/mL (Abcam plc, Cambridge, UK) followed by incubation with secondary antibody, (Goat anti-rabbit polyclonal antibody, 1:600, Beyotime Institute of Biotechnology) for 1 h at 37oC. The immunoreactivity was detected using 3,3´diaminobenzidine tetrahydrochloride (DAB substrate kit, Vector Labs, Santa Cruz Biotechnology Inc.) was used as the chromogen. The section was counterstained with hematoxylin (Beyotime Institute of Biotechnology). β-AR-positive staining in the cortex of the brain and the atrium of the heart was identified by the presence of brown staining of cells specifically present around the cytoplasm and imaged with a Camera-probed (Toupcam Industrial Digital Camera, model no. UCMOSO5100KPA, made in China) phase contrast microscope (CKX41747, Olympus Corporation Tokyo, Japan). The staining intensity of the sections was analyzed using the Image-Pro plus 6.0 image analysis system (Media Cybernetics, Inc., Rockville, MD, USA) in brains and hearts of rats induced with CI/RI and statistically computed using SPSS.

## Slide 11
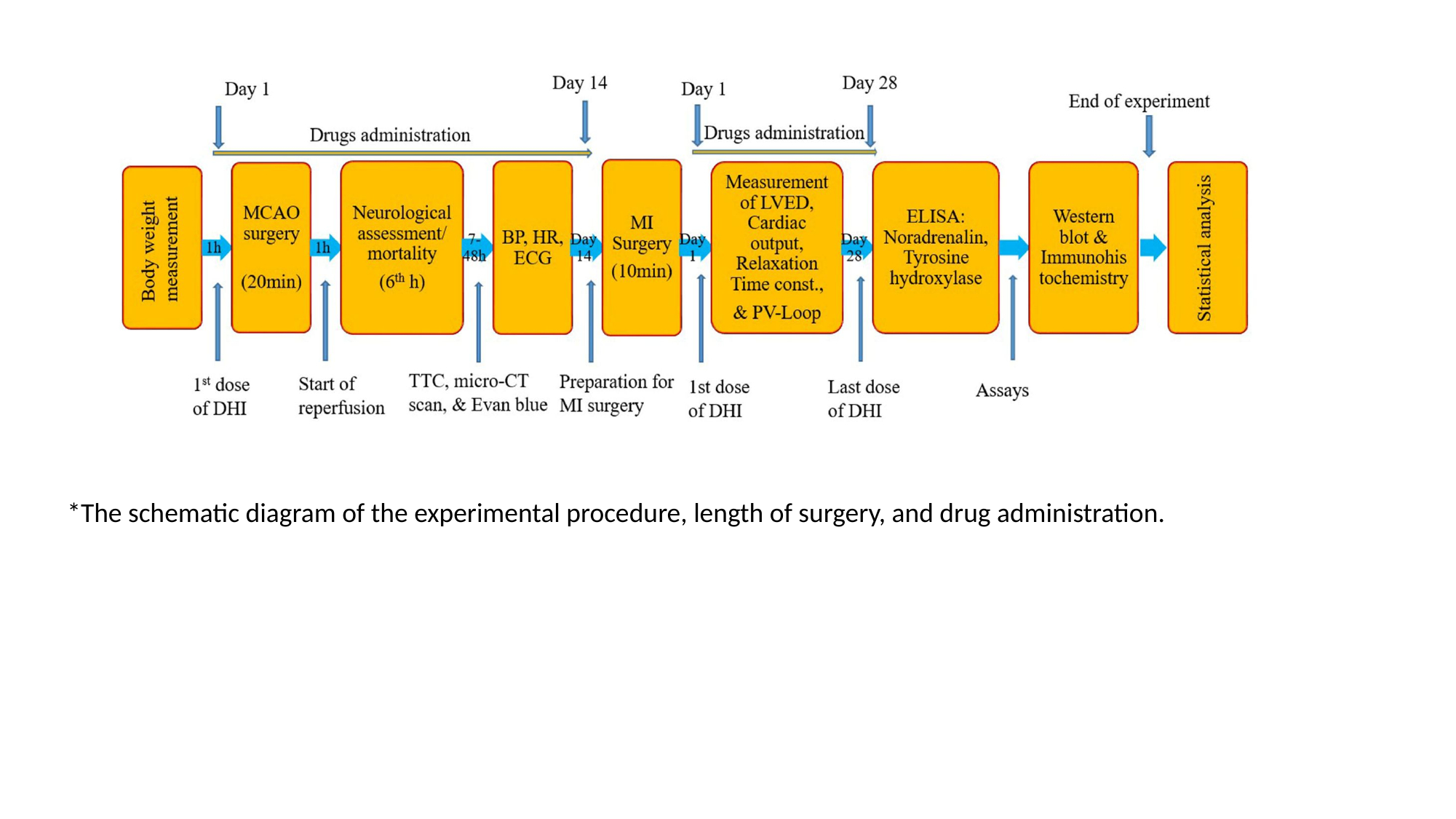

*The schematic diagram of the experimental procedure, length of surgery, and drug administration.

## Slide 12
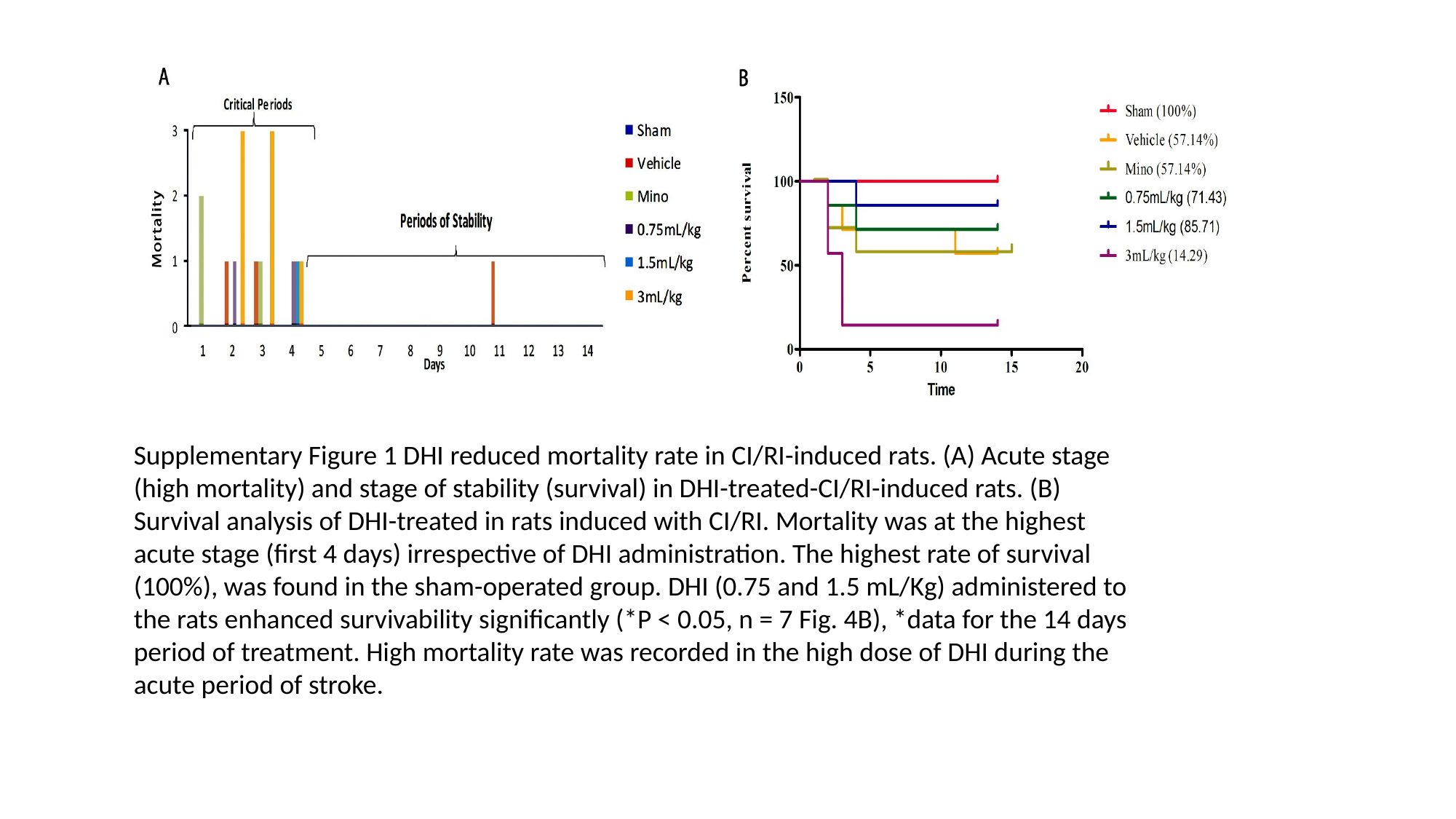

Supplementary Figure 1 DHI reduced mortality rate in CI/RI-induced rats. (A) Acute stage (high mortality) and stage of stability (survival) in DHI-treated-CI/RI-induced rats. (B) Survival analysis of DHI-treated in rats induced with CI/RI. Mortality was at the highest acute stage (first 4 days) irrespective of DHI administration. The highest rate of survival (100%), was found in the sham-operated group. DHI (0.75 and 1.5 mL/Kg) administered to the rats enhanced survivability significantly (*P < 0.05, n = 7 Fig. 4B), *data for the 14 days period of treatment. High mortality rate was recorded in the high dose of DHI during the acute period of stroke.

## Slide 13
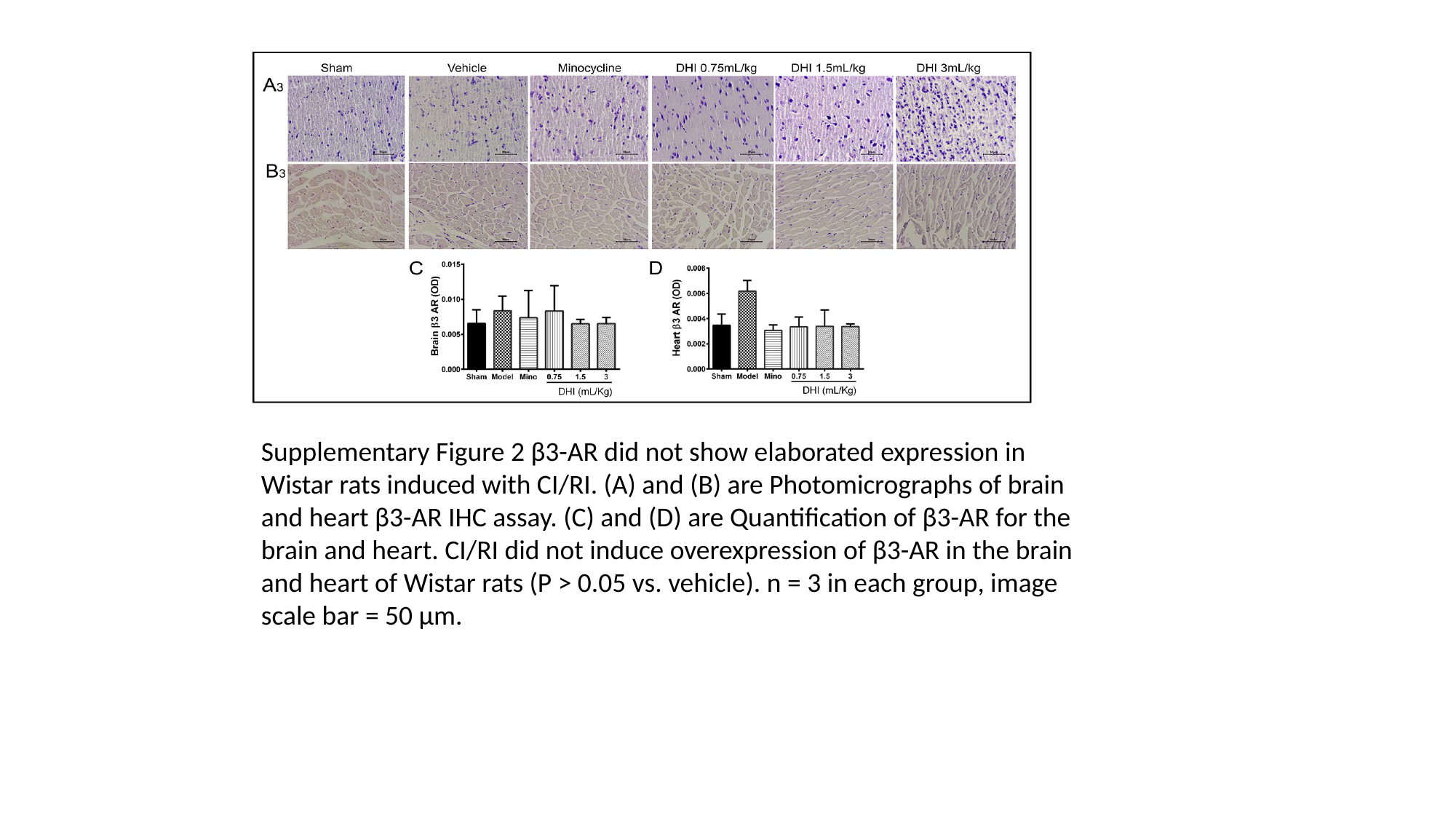

Supplementary Figure 2 β3-AR did not show elaborated expression in Wistar rats induced with CI/RI. (A) and (B) are Photomicrographs of brain and heart β3-AR IHC assay. (C) and (D) are Quantification of β3-AR for the brain and heart. CI/RI did not induce overexpression of β3-AR in the brain and heart of Wistar rats (P > 0.05 vs. vehicle). n = 3 in each group, image scale bar = 50 µm.

## Slide 14
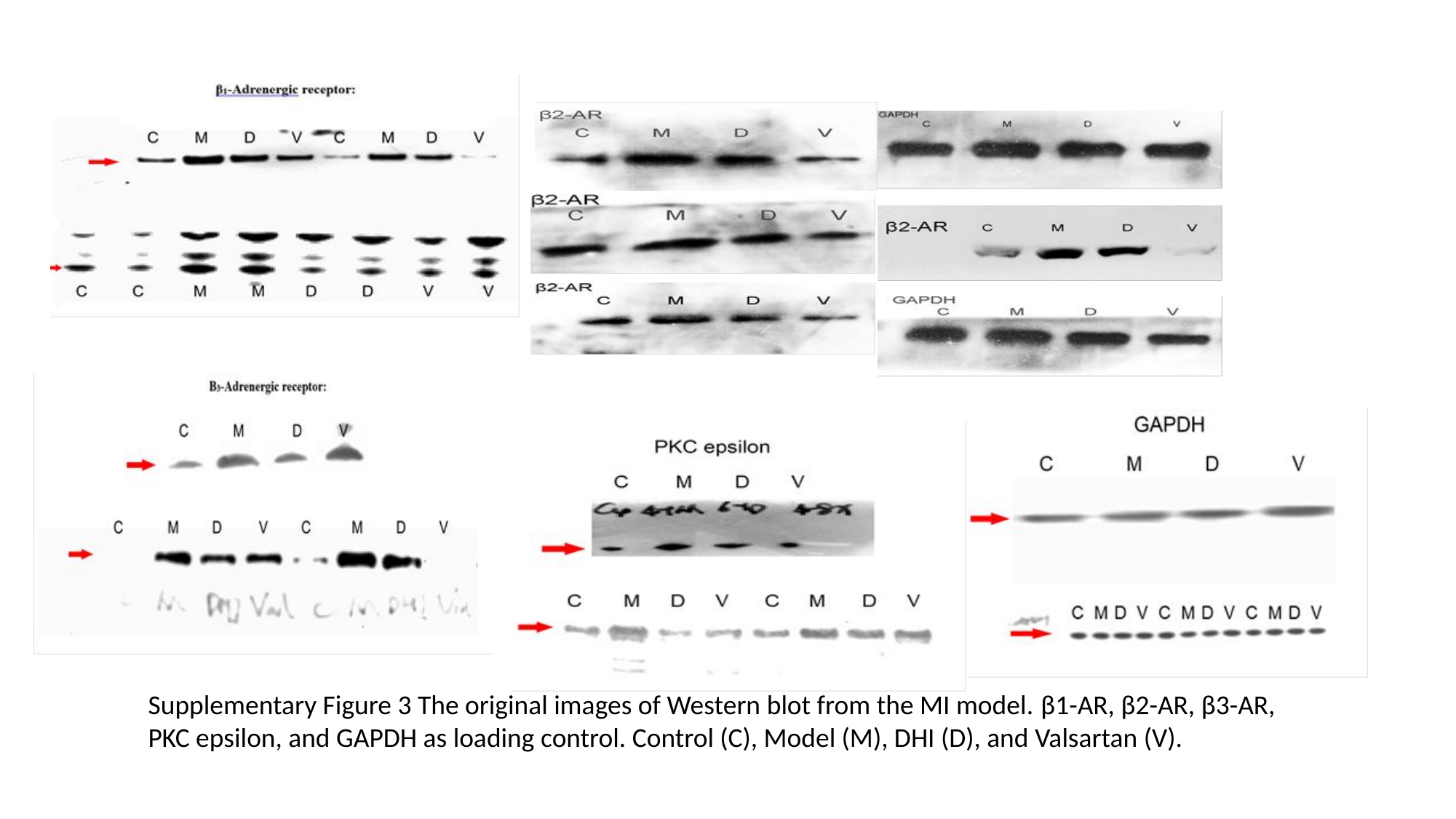

Supplementary Figure 3 The original images of Western blot from the MI model. β1-AR, β2-AR, β3-AR, PKC epsilon, and GAPDH as loading control. Control (C), Model (M), DHI (D), and Valsartan (V).

## Slide 15
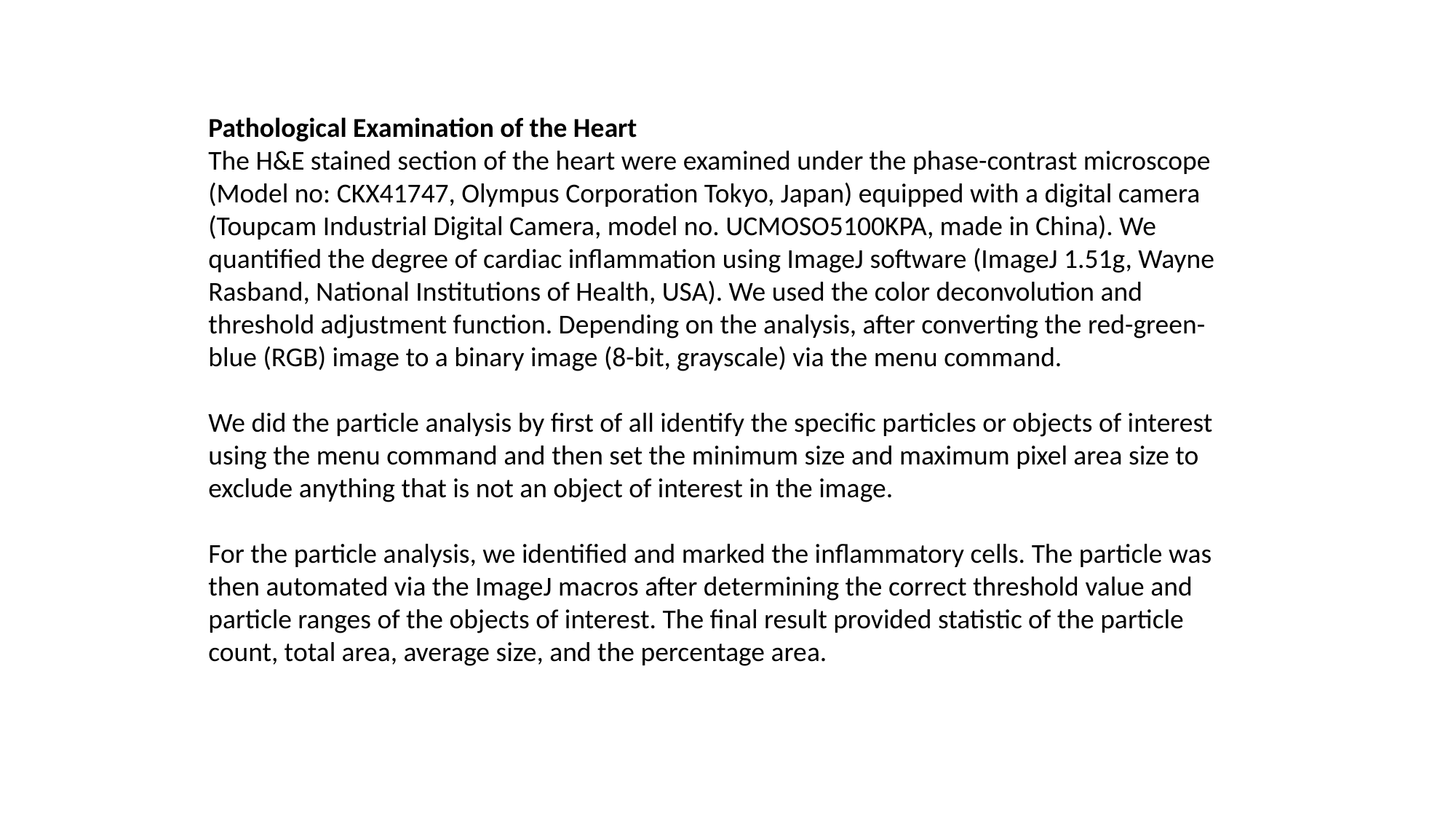

Pathological Examination of the Heart
The H&E stained section of the heart were examined under the phase-contrast microscope (Model no: CKX41747, Olympus Corporation Tokyo, Japan) equipped with a digital camera (Toupcam Industrial Digital Camera, model no. UCMOSO5100KPA, made in China). We quantified the degree of cardiac inflammation using ImageJ software (ImageJ 1.51g, Wayne Rasband, National Institutions of Health, USA). We used the color deconvolution and threshold adjustment function. Depending on the analysis, after converting the red-green-blue (RGB) image to a binary image (8-bit, grayscale) via the menu command.
We did the particle analysis by first of all identify the specific particles or objects of interest using the menu command and then set the minimum size and maximum pixel area size to exclude anything that is not an object of interest in the image.
For the particle analysis, we identified and marked the inflammatory cells. The particle was then automated via the ImageJ macros after determining the correct threshold value and particle ranges of the objects of interest. The final result provided statistic of the particle count, total area, average size, and the percentage area.

## Slide 16
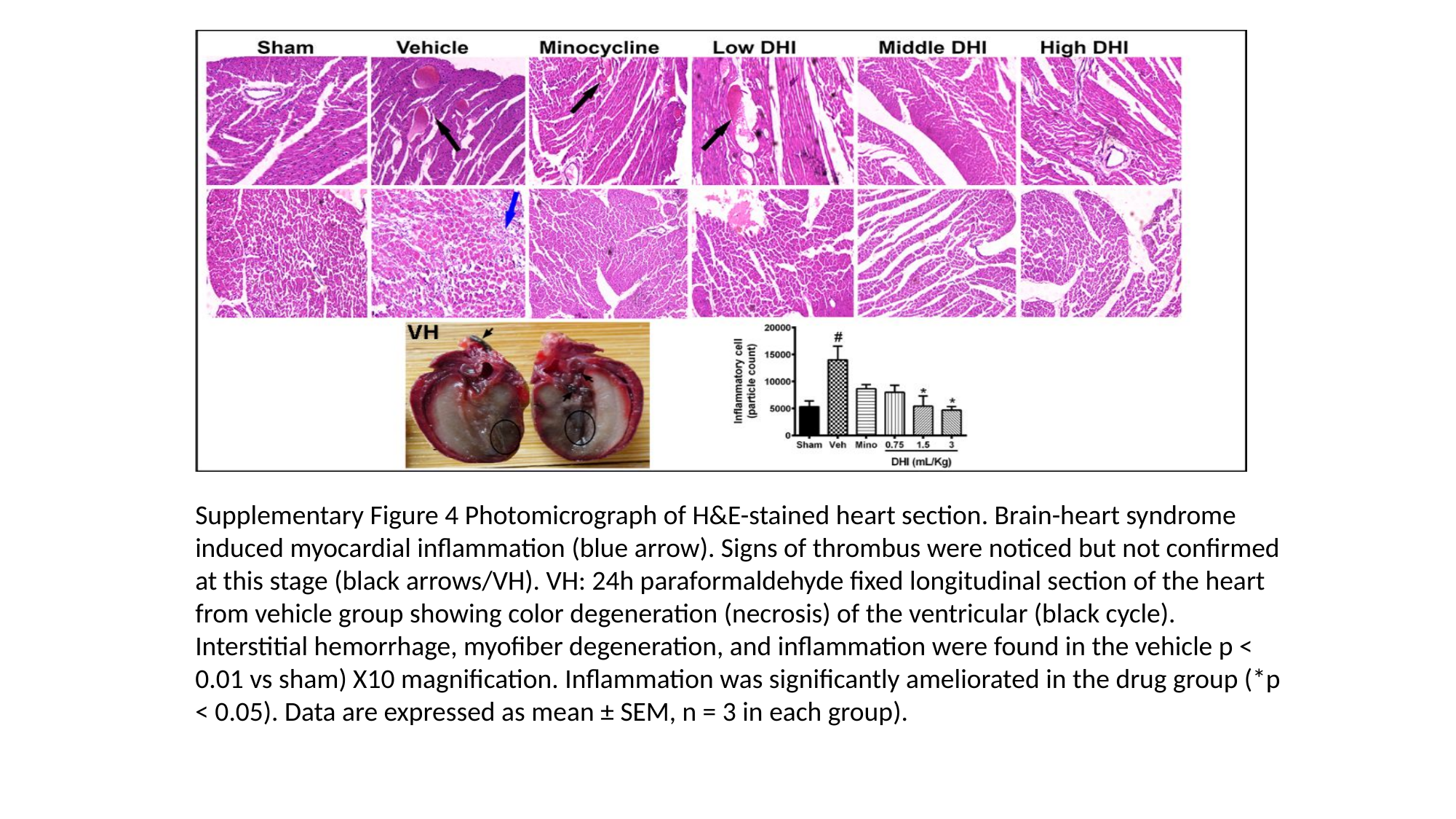

Supplementary Figure 4 Photomicrograph of H&E-stained heart section. Brain-heart syndrome induced myocardial inflammation (blue arrow). Signs of thrombus were noticed but not confirmed at this stage (black arrows/VH). VH: 24h paraformaldehyde fixed longitudinal section of the heart from vehicle group showing color degeneration (necrosis) of the ventricular (black cycle). Interstitial hemorrhage, myofiber degeneration, and inflammation were found in the vehicle p < 0.01 vs sham) X10 magnification. Inflammation was significantly ameliorated in the drug group (*p < 0.05). Data are expressed as mean ± SEM, n = 3 in each group).

## Slide 17
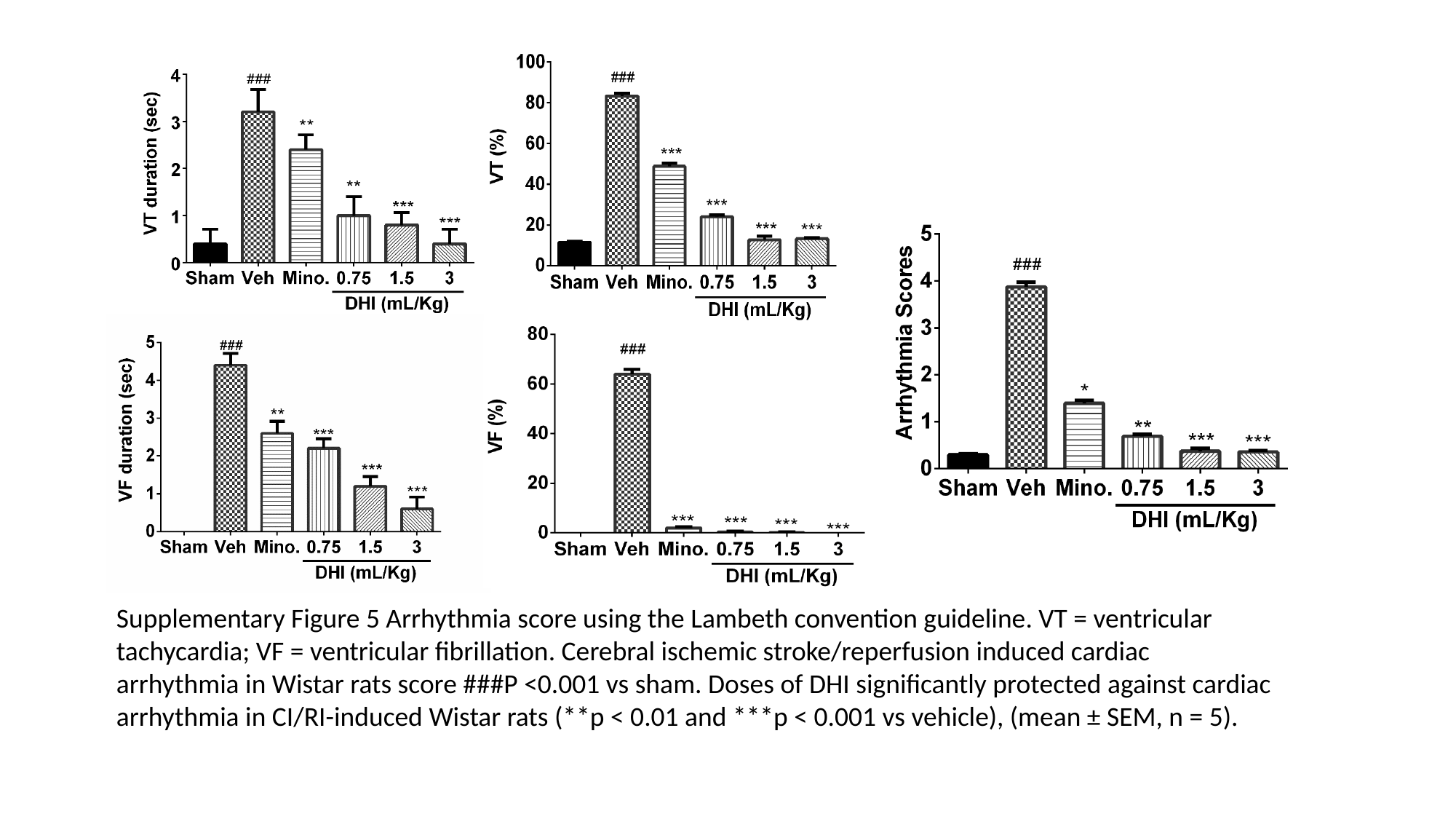

Supplementary Figure 5 Arrhythmia score using the Lambeth convention guideline. VT = ventricular tachycardia; VF = ventricular fibrillation. Cerebral ischemic stroke/reperfusion induced cardiac arrhythmia in Wistar rats score ###P <0.001 vs sham. Doses of DHI significantly protected against cardiac arrhythmia in CI/RI-induced Wistar rats (**p < 0.01 and ***p < 0.001 vs vehicle), (mean ± SEM, n = 5).

## Slide 18
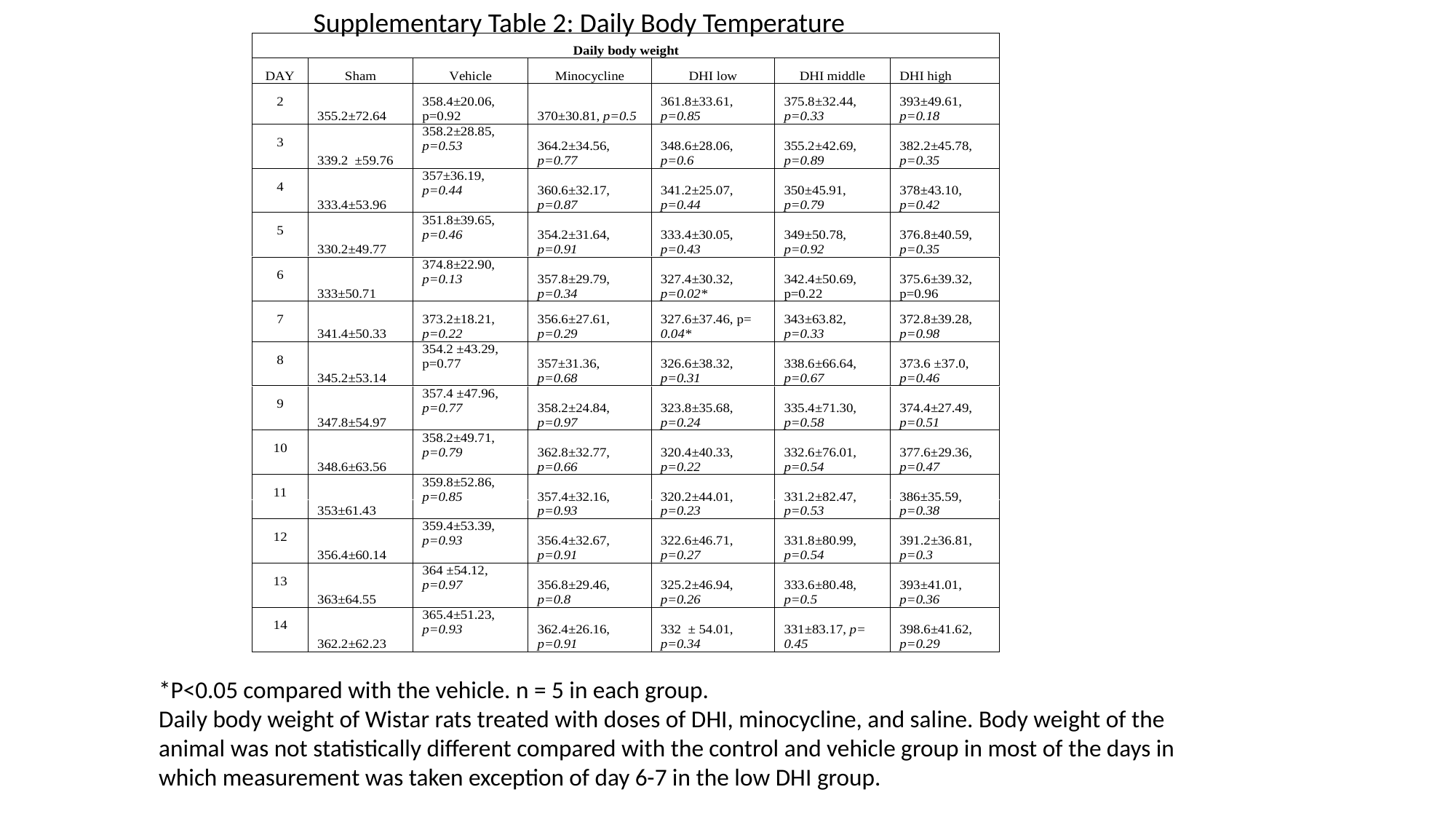

Supplementary Table 2: Daily Body Temperature
*P<0.05 compared with the vehicle. n = 5 in each group.
Daily body weight of Wistar rats treated with doses of DHI, minocycline, and saline. Body weight of the animal was not statistically different compared with the control and vehicle group in most of the days in which measurement was taken exception of day 6-7 in the low DHI group.

## Slide 19
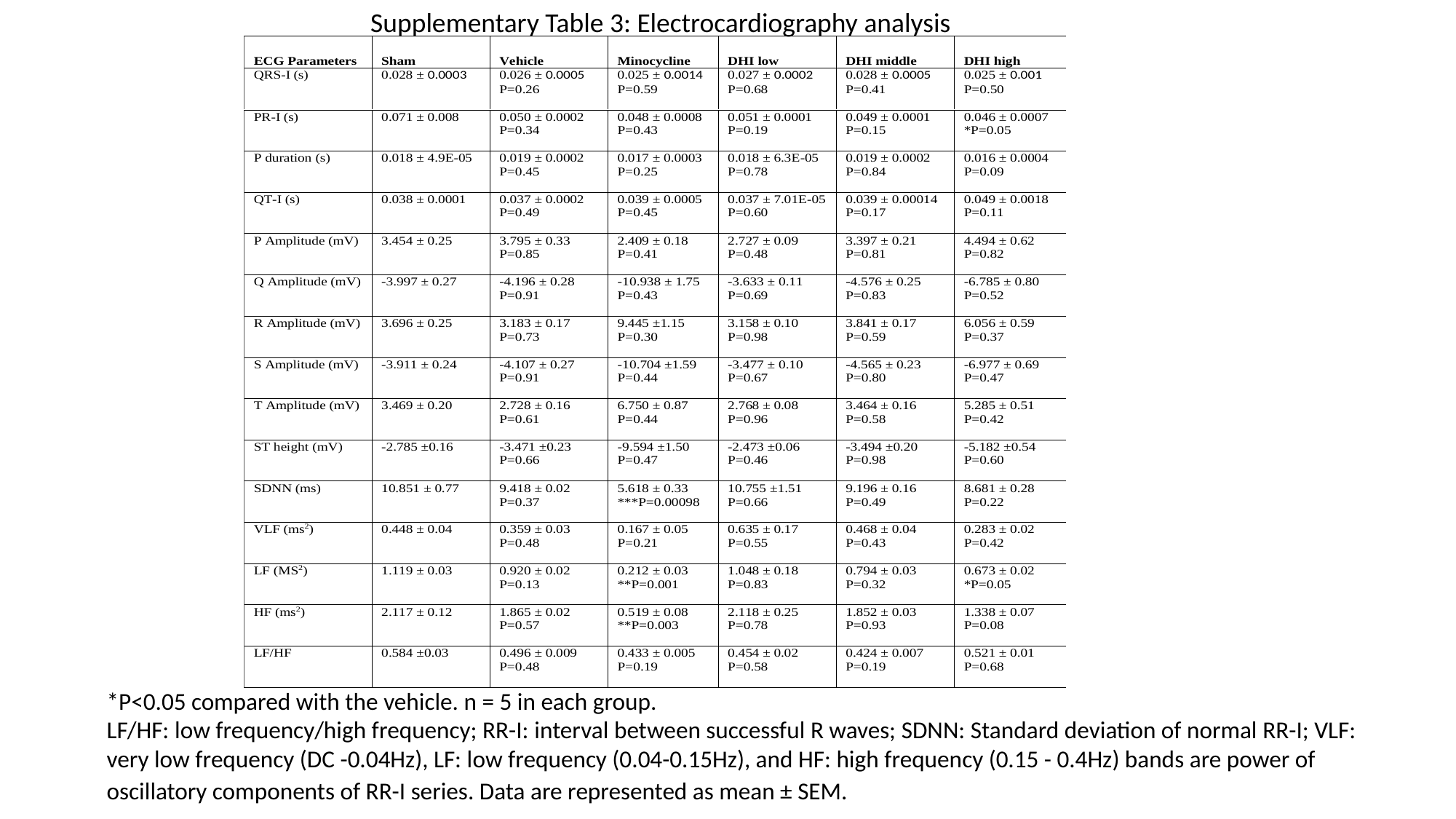

Supplementary Table 3: Electrocardiography analysis
*P<0.05 compared with the vehicle. n = 5 in each group.
LF/HF: low frequency/high frequency; RR-I: interval between successful R waves; SDNN: Standard deviation of normal RR-I; VLF: very low frequency (DC -0.04Hz), LF: low frequency (0.04-0.15Hz), and HF: high frequency (0.15 - 0.4Hz) bands are power of oscillatory components of RR-I series. Data are represented as mean ± SEM.

## Slide 20
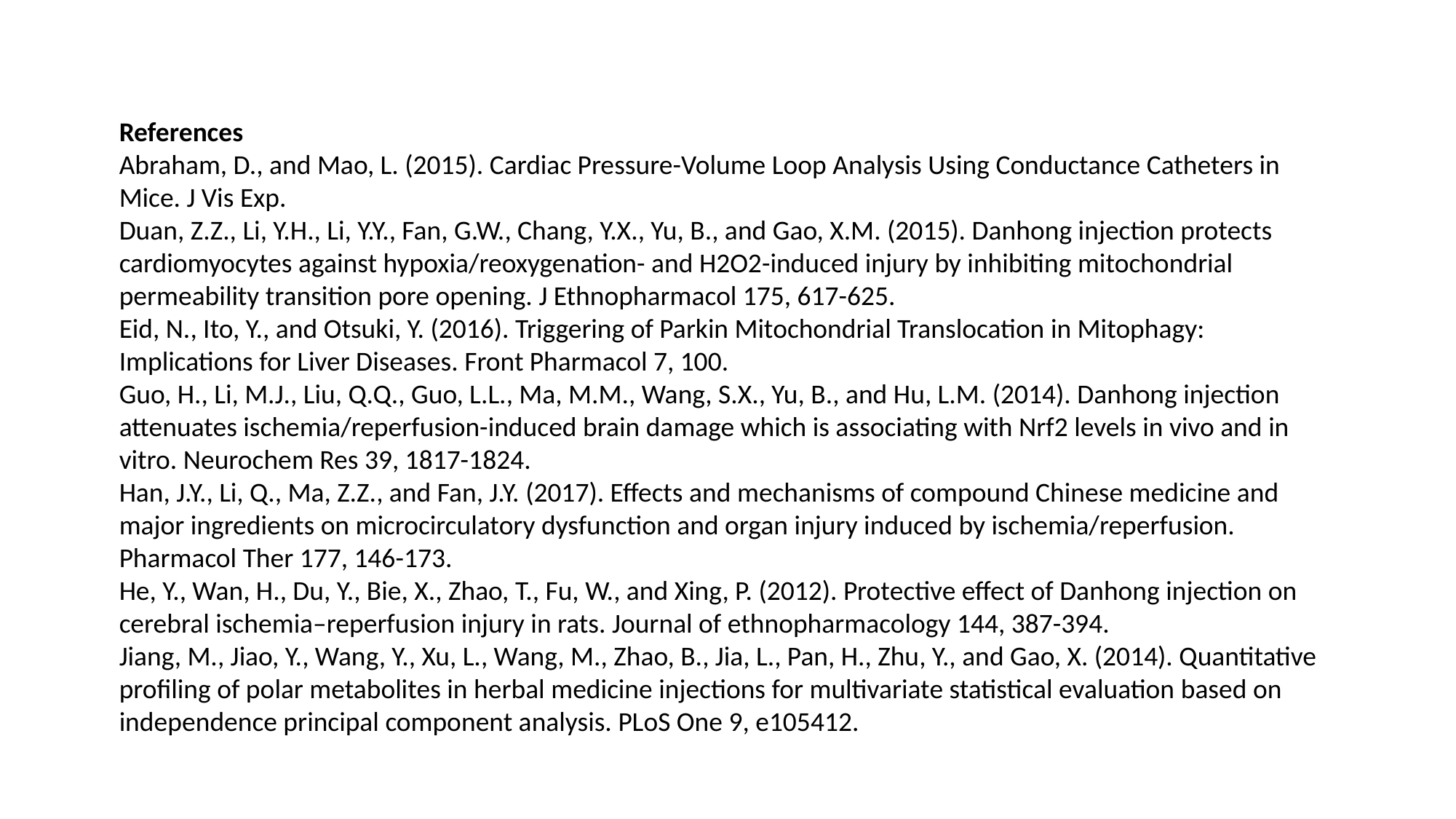

References
Abraham, D., and Mao, L. (2015). Cardiac Pressure-Volume Loop Analysis Using Conductance Catheters in Mice. J Vis Exp.
Duan, Z.Z., Li, Y.H., Li, Y.Y., Fan, G.W., Chang, Y.X., Yu, B., and Gao, X.M. (2015). Danhong injection protects cardiomyocytes against hypoxia/reoxygenation- and H2O2-induced injury by inhibiting mitochondrial permeability transition pore opening. J Ethnopharmacol 175, 617-625.
Eid, N., Ito, Y., and Otsuki, Y. (2016). Triggering of Parkin Mitochondrial Translocation in Mitophagy: Implications for Liver Diseases. Front Pharmacol 7, 100.
Guo, H., Li, M.J., Liu, Q.Q., Guo, L.L., Ma, M.M., Wang, S.X., Yu, B., and Hu, L.M. (2014). Danhong injection attenuates ischemia/reperfusion-induced brain damage which is associating with Nrf2 levels in vivo and in vitro. Neurochem Res 39, 1817-1824.
Han, J.Y., Li, Q., Ma, Z.Z., and Fan, J.Y. (2017). Effects and mechanisms of compound Chinese medicine and major ingredients on microcirculatory dysfunction and organ injury induced by ischemia/reperfusion. Pharmacol Ther 177, 146-173.
He, Y., Wan, H., Du, Y., Bie, X., Zhao, T., Fu, W., and Xing, P. (2012). Protective effect of Danhong injection on cerebral ischemia–reperfusion injury in rats. Journal of ethnopharmacology 144, 387-394.
Jiang, M., Jiao, Y., Wang, Y., Xu, L., Wang, M., Zhao, B., Jia, L., Pan, H., Zhu, Y., and Gao, X. (2014). Quantitative profiling of polar metabolites in herbal medicine injections for multivariate statistical evaluation based on independence principal component analysis. PLoS One 9, e105412.

## Slide 21
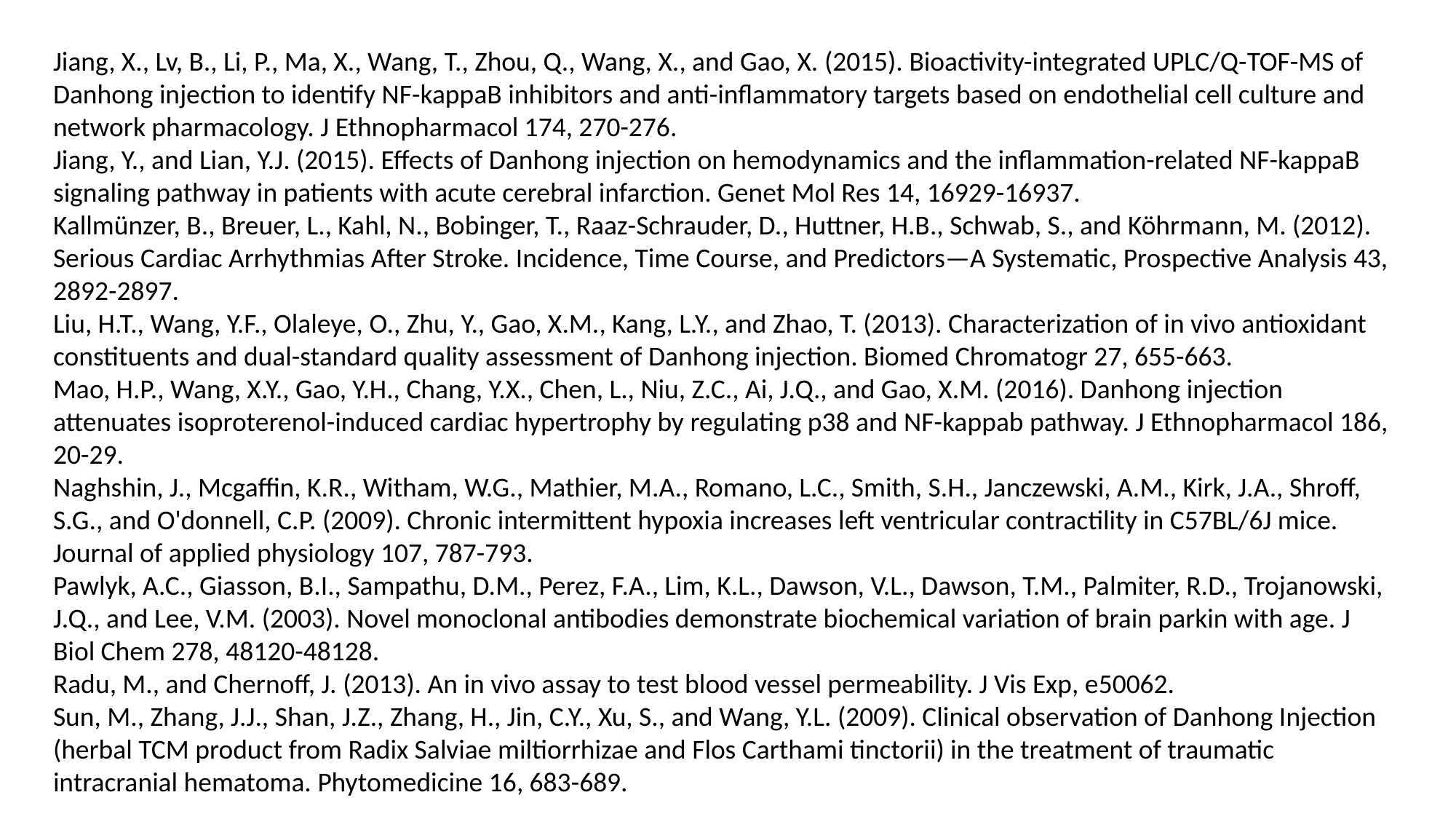

Jiang, X., Lv, B., Li, P., Ma, X., Wang, T., Zhou, Q., Wang, X., and Gao, X. (2015). Bioactivity-integrated UPLC/Q-TOF-MS of Danhong injection to identify NF-kappaB inhibitors and anti-inflammatory targets based on endothelial cell culture and network pharmacology. J Ethnopharmacol 174, 270-276.
Jiang, Y., and Lian, Y.J. (2015). Effects of Danhong injection on hemodynamics and the inflammation-related NF-kappaB signaling pathway in patients with acute cerebral infarction. Genet Mol Res 14, 16929-16937.
Kallmünzer, B., Breuer, L., Kahl, N., Bobinger, T., Raaz-Schrauder, D., Huttner, H.B., Schwab, S., and Köhrmann, M. (2012). Serious Cardiac Arrhythmias After Stroke. Incidence, Time Course, and Predictors—A Systematic, Prospective Analysis 43, 2892-2897.
Liu, H.T., Wang, Y.F., Olaleye, O., Zhu, Y., Gao, X.M., Kang, L.Y., and Zhao, T. (2013). Characterization of in vivo antioxidant constituents and dual-standard quality assessment of Danhong injection. Biomed Chromatogr 27, 655-663.
Mao, H.P., Wang, X.Y., Gao, Y.H., Chang, Y.X., Chen, L., Niu, Z.C., Ai, J.Q., and Gao, X.M. (2016). Danhong injection attenuates isoproterenol-induced cardiac hypertrophy by regulating p38 and NF-kappab pathway. J Ethnopharmacol 186, 20-29.
Naghshin, J., Mcgaffin, K.R., Witham, W.G., Mathier, M.A., Romano, L.C., Smith, S.H., Janczewski, A.M., Kirk, J.A., Shroff, S.G., and O'donnell, C.P. (2009). Chronic intermittent hypoxia increases left ventricular contractility in C57BL/6J mice. Journal of applied physiology 107, 787-793.
Pawlyk, A.C., Giasson, B.I., Sampathu, D.M., Perez, F.A., Lim, K.L., Dawson, V.L., Dawson, T.M., Palmiter, R.D., Trojanowski, J.Q., and Lee, V.M. (2003). Novel monoclonal antibodies demonstrate biochemical variation of brain parkin with age. J Biol Chem 278, 48120-48128.
Radu, M., and Chernoff, J. (2013). An in vivo assay to test blood vessel permeability. J Vis Exp, e50062.
Sun, M., Zhang, J.J., Shan, J.Z., Zhang, H., Jin, C.Y., Xu, S., and Wang, Y.L. (2009). Clinical observation of Danhong Injection (herbal TCM product from Radix Salviae miltiorrhizae and Flos Carthami tinctorii) in the treatment of traumatic intracranial hematoma. Phytomedicine 16, 683-689.

## Slide 22
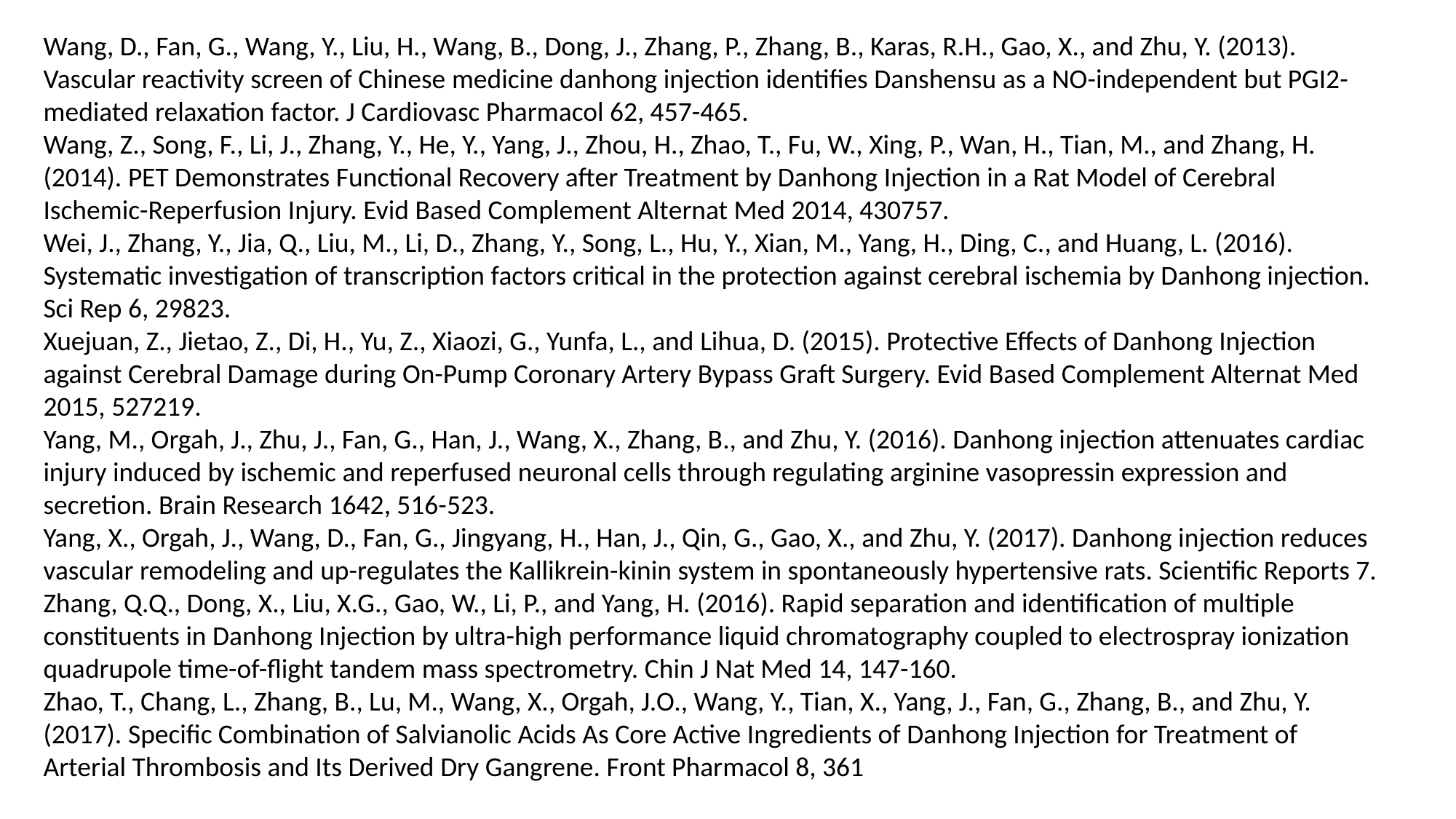

Wang, D., Fan, G., Wang, Y., Liu, H., Wang, B., Dong, J., Zhang, P., Zhang, B., Karas, R.H., Gao, X., and Zhu, Y. (2013). Vascular reactivity screen of Chinese medicine danhong injection identifies Danshensu as a NO-independent but PGI2-mediated relaxation factor. J Cardiovasc Pharmacol 62, 457-465.
Wang, Z., Song, F., Li, J., Zhang, Y., He, Y., Yang, J., Zhou, H., Zhao, T., Fu, W., Xing, P., Wan, H., Tian, M., and Zhang, H. (2014). PET Demonstrates Functional Recovery after Treatment by Danhong Injection in a Rat Model of Cerebral Ischemic-Reperfusion Injury. Evid Based Complement Alternat Med 2014, 430757.
Wei, J., Zhang, Y., Jia, Q., Liu, M., Li, D., Zhang, Y., Song, L., Hu, Y., Xian, M., Yang, H., Ding, C., and Huang, L. (2016). Systematic investigation of transcription factors critical in the protection against cerebral ischemia by Danhong injection. Sci Rep 6, 29823.
Xuejuan, Z., Jietao, Z., Di, H., Yu, Z., Xiaozi, G., Yunfa, L., and Lihua, D. (2015). Protective Effects of Danhong Injection against Cerebral Damage during On-Pump Coronary Artery Bypass Graft Surgery. Evid Based Complement Alternat Med 2015, 527219.
Yang, M., Orgah, J., Zhu, J., Fan, G., Han, J., Wang, X., Zhang, B., and Zhu, Y. (2016). Danhong injection attenuates cardiac injury induced by ischemic and reperfused neuronal cells through regulating arginine vasopressin expression and secretion. Brain Research 1642, 516-523.
Yang, X., Orgah, J., Wang, D., Fan, G., Jingyang, H., Han, J., Qin, G., Gao, X., and Zhu, Y. (2017). Danhong injection reduces vascular remodeling and up-regulates the Kallikrein-kinin system in spontaneously hypertensive rats. Scientific Reports 7.
Zhang, Q.Q., Dong, X., Liu, X.G., Gao, W., Li, P., and Yang, H. (2016). Rapid separation and identification of multiple constituents in Danhong Injection by ultra-high performance liquid chromatography coupled to electrospray ionization quadrupole time-of-flight tandem mass spectrometry. Chin J Nat Med 14, 147-160.
Zhao, T., Chang, L., Zhang, B., Lu, M., Wang, X., Orgah, J.O., Wang, Y., Tian, X., Yang, J., Fan, G., Zhang, B., and Zhu, Y. (2017). Specific Combination of Salvianolic Acids As Core Active Ingredients of Danhong Injection for Treatment of Arterial Thrombosis and Its Derived Dry Gangrene. Front Pharmacol 8, 361.
Wang, D., Fan, G., Wang, Y., Liu, H., Wang, B., Dong, J., Zhang, P., Zhang, B., Karas, R.H., Gao, X., and Zhu, Y. (2013). Vascular reactivity screen of Chinese medicine danhong injection identifies Danshensu as a NO-independent but PGI2-mediated relaxation factor. J Cardiovasc Pharmacol 62, 457-465.
Wang, Z., Song, F., Li, J., Zhang, Y., He, Y., Yang, J., Zhou, H., Zhao, T., Fu, W., Xing, P., Wan, H., Tian, M., and Zhang, H. (2014). PET Demonstrates Functional Recovery after Treatment by Danhong Injection in a Rat Model of Cerebral Ischemic-Reperfusion Injury. Evid Based Complement Alternat Med 2014, 430757.
Wei, J., Zhang, Y., Jia, Q., Liu, M., Li, D., Zhang, Y., Song, L., Hu, Y., Xian, M., Yang, H., Ding, C., and Huang, L. (2016). Systematic investigation of transcription factors critical in the protection against cerebral ischemia by Danhong injection. Sci Rep 6, 29823.
Xuejuan, Z., Jietao, Z., Di, H., Yu, Z., Xiaozi, G., Yunfa, L., and Lihua, D. (2015). Protective Effects of Danhong Injection against Cerebral Damage during On-Pump Coronary Artery Bypass Graft Surgery. Evid Based Complement Alternat Med 2015, 527219.
Yang, M., Orgah, J., Zhu, J., Fan, G., Han, J., Wang, X., Zhang, B., and Zhu, Y. (2016). Danhong injection attenuates cardiac injury induced by ischemic and reperfused neuronal cells through regulating arginine vasopressin expression and secretion. Brain Research 1642, 516-523.
Yang, X., Orgah, J., Wang, D., Fan, G., Jingyang, H., Han, J., Qin, G., Gao, X., and Zhu, Y. (2017). Danhong injection reduces vascular remodeling and up-regulates the Kallikrein-kinin system in spontaneously hypertensive rats. Scientific Reports 7.
Zhang, Q.Q., Dong, X., Liu, X.G., Gao, W., Li, P., and Yang, H. (2016). Rapid separation and identification of multiple constituents in Danhong Injection by ultra-high performance liquid chromatography coupled to electrospray ionization quadrupole time-of-flight tandem mass spectrometry. Chin J Nat Med 14, 147-160.
Zhao, T., Chang, L., Zhang, B., Lu, M., Wang, X., Orgah, J.O., Wang, Y., Tian, X., Yang, J., Fan, G., Zhang, B., and Zhu, Y. (2017). Specific Combination of Salvianolic Acids As Core Active Ingredients of Danhong Injection for Treatment of Arterial Thrombosis and Its Derived Dry Gangrene. Front Pharmacol 8, 361
